# Endophytic Fungus Isolated From *Achyrocline satureioides* Exhibits Selective Antiglioma Activity—The Role of Sch-642305

**DOI:** 10.3389/fonc.2018.00476

**Published:** 2018-10-29

**Authors:** Nathalia Stark Pedra, Kennia de Cássia Araújo Galdino, Daniel Schuch da Silva, Priscila Treptow Ramos, Natália Pontes Bona, Mayara Sandrielly Pereira Soares, Juliana Hoffstater Azambuja, Kirley Marques Canuto, Edy Sousa de Brito, Paulo Riceli Vasconcelos Ribeiro, Ana Sheila de Queiroz Souza, Wilson Cunico, Francieli Moro Stefanello, Roselia Maria Spanevello, Elizandra Braganhol

**Affiliations:** ^1^Programa de Pós-Graduação em Bioquímica e Bioprospecção, Centro de Ciências Químicas, Farmacêuticas e de Alimentos, Universidade Federal de Pelotas, Pelotas, Brazil; ^2^Departamento de Ciências Básicas da Saúde, Universidade Federal de Ciências da Saúde de Porto Alegre, Porto Alegre, Brazil; ^3^Embrapa Agroindústria Tropical, Fortaleza, Brazil

**Keywords:** endophytic fungus, *Achyrocline satureioides*, glioblastoma, lactone, antineoplasic agent, antioxidant

## Abstract

Glioblastoma is the most devastating primary brain tumor. Current treatment is palliative, making necessary the development of new therapeutic strategies to offer alternatives to patients. Therefore, endophytes represent an interesting source of natural metabolites with anticancer potential. These microorganisms reside in tissues of living plants and act to improve their growth. Evidence revealed that several medicinal plants are colonized by endophytic fungi producer of antitumor metabolites. *Achyrocline satureioides* is a Brazilian medicinal plant characterized by its properties against gastrointestinal disturbances, anticancer and antioxidant effects. However, there are no reports describing the endophytic composition of *A. satureioides*. The present study proposes the isolation of endophytic fungus from *A. satureioides*, extract preparation, phytochemical characterization and evaluation of its antiglioma potential. Our data showed that crude extracts of endophyte decreased glioma viability with IC_50_ values of 1.60–1.63 μg/mL to eDCM (dichloromethane extract) and 37.30–55.12 μg/mL to eEtAc (ethyl acetate extract), respectively. Crude extracts induced cell death by apoptosis with modulation of redox status. In order to bioprospect anticancer metabolites, endophytic fungus extracts were subjected to guided fractionation and purification yielded five fractions of each extract. Six of ten fractions showed selective antiproliferative activity against glioma cells, with IC_50_ values ranged from 0.95 to 131.3 μg/mL. F3_DCM_ (from eDCM) and F3_EtAc_ (from eEtAc) fractions promoted C6 glioma toxicity with IC_50_ of 1.0 and 27.05 μg/mL, respectively. F3_EtAc_ fraction induced late apoptosis and arrest in G2/M stage, while F3_DCM_ promoted apoptosis with arrest in Sub-G1 phase. Moreover, F3_DCM_ increased antioxidant defense and decreased ROS production. Additionally, F3_DCM_ showed no cytotoxic activity against astrocytes, revealing selective effect. Based on promising potential of F3_DCM_, we identified the production of Sch-642305, a lactone, which showed antiproliferative properties with IC_50_ values of 1.1 and 7.6 μg/mL to C6 and U138MG gliomas, respectively. Sch-642305 promoted arrest on cell cycle in G2/M inducing apoptosis. Furthermore, this lactone decreased glioma cell migration and modulated redox status, increasing superoxide dismutase and catalase activities and enhancing sulfhydryl content, consequently suppressing reactive species of oxygen generation. Taken together, these results indicate that metabolites produced by endophytic fungus isolated from *A. satureioides* have therapeutic potential as antiglioma agent.

## Introduction

Glioblastoma, a grade IV glioma, is the most malignant type of brain cancer characterized by high cell heterogeneity, diffuse brain infiltration, necrosis, high rate of cell proliferation, and resistance to current treatments (1, 2). Despite aggressive, multimodal therapy consisting of surgery, radiation, and chemotherapy, the outcome of patients with glioblastoma remains poor (3) with median overall survival time of ~15–17 months (4). The maintenance of redox homeostasis is crucial for normal cell physiology and reactive oxygen species (ROS) are known to regulate cellular events, including cell growth, differentiation, apoptosis, metabolism and others (5). Several hallmarks of cancer associated with neoplastic growth promote increased ROS levels inducing elevated oxidative stress (6). This cellular redox imbalance has been found in glioblastoma. High quantities of ROS into the cells can react with macromolecules, including chromosomal and mitochondrial DNA, leading to damage and malfunction of DNA repair enzymes (7). Therefore, new treatments able to modulate this redox status may be a feasible therapeutic approach against glioblastoma.

Natural products have been exploited extensively to new pharmaceuticals development to treat several diseases. *A. satureioides* Lam. (DC) (Asteraceae) popularly known as “marcela” have received particular attention for their pharmacological activities (8). This plant is native medicinal herb in South America, used in Brazilian folk medicine as an analgesic, sedative, anti-inflammatory and mainly to treat gastrointestinal disorders (9, 10). Plants are continuously involved in crosstalk with endophytic microorganisms leading to the selection of specific functional traits (11). Indeed, endophytic fungi produce a variety of bioactive metabolites that may directly or indirectly be used as therapeutic agents (12–14). These microorganisms have also been found to produce the same important natural products synthesized by the host plant, such as alkaloids, phenols, coumarins, steroids, terpenoids, peptides and others with anticancer properties (15). Although the chemical constituents and the biological properties of genus *Achyrocline* have been extensively studied (16–18), there are no evidence about the endophytic fungi associated with this genus and the possible therapeutic activities of these microorganisms. Additionally, considering the role of redox status in glioblastoma aggressiveness and how this imbalance contribute to gliomagenesis (7), it becomes important the investigation of new therapeutic agents that modulate redox status. Therefore, in present study we evaluated the selective antiglioma activity of crude organic and fractionated extracts of endophytic fungus from *A. satureioides* and their effects in the modulation of redox environment on glioblastoma through evaluation of oxidative stress biomarkers. Additionally, phytochemical characterization was performed and the macrolide (macrocyclic lactone) Sch-642305 was identified as one of the bioactive molecules with promising antiglioma activity produced by endophytic fungus from *A. satureioides*.

## Materials and methods

### Chemicals

Dulbecco's modified Eagle's medium (DMEM); fungizone; penicillin/streptomycin; 0.5%trypsin/EDTA solution and fetal bovine serum (FBS) were obtained from Gibco (Gibco BRL, Carlsbad, CA, United States). 4-(2-Hydroxyethyl)piperazine-1-ethanesulfonic acid (HEPES); sodium bicarbonate (NaHCO_3_), Dimethylsulphoxide (DMSO); 3(4, 5-dimethyl)-2,5diphenyl tetrazolium bromide (MTT) were purchased from Sigma Chemical Co. (St. Louis, MO, United States). Trichloroacetic acid and hydrogen peroxide were purchased from Synth® (Brazil). All other chemicals and solvents used were of analytical grade. Agar and dextrose was provided by Dinâmica (Dinâmica Química Ltda, Diadema, SP, BR).

### Collection of plant tissue and isolation of endophytic fungi

Stems of *A. satureioides* (Lam.) D.C. were collected at Transbrasiliana Highway (Rio Grande do Sul, Brazil; geographic coordinates: 31°44′34.7″S and 54°09′19.2″W) and it was identified by Dra. Raquel Ludke from the Botany Department (Biology Institute, UFPel), and a voucher specimen was deposited under the code PEL N° 21079. Surface sterilization of healthy stems was performed according Bertozzo and Machado (19), with some modifications. Briefly, tissue material was thoroughly washed using distilled water, sterilized with 70% ethanol for 30 s and 2% sodium hypochlorite for 30 min, then rinsed with sterile distilled water for three times to accomplish surface sterilization. Next, samples were cut into 6–8 pieces (6–10 mm in size), placed on water-agar medium and incubated at 25 ± 2°C under controlled light conditions (Thelga; Dom Bosco, MG, BR). Following 7 days of culture, hyphal tips of fungi that emerged was periodically picked on petri plates containing 1.7% PDA (potato-dextrose-agar) medium for purification and maintained at same conditions described above. Stock cultures were stored at 25 ± 2°C and maintained in the culture collection of NeuroCan Laboratory (UFPel).

### Morphological identification of endophytic fungus

Isolated fungi were observed and identified at the genus level by culture and microscopic characters of asexual/sexual spores, according Rocha et al. (20) with modifications. Briefly, endophytic fungus was seeded in 500 μL of PDA medium distributed on a slide held inside petri dish containing a filter paper soaked in sterile distilled water to maintain the moisture of the system for 20 days at 25°C. After that, the endophytic fungus was stained with cotton blue to identify its morphology under light microscopy. The identification was based on published descriptions.

### Preparation of crude extracts

The endophytic strain was cultivated on 1.7% PDA medium at 25 ± 2°C under controlled light conditions. Then plugs of mycelium (about 8 mm diameter) from the edges of 7-day-old cultures were cut and inoculated aseptically into a 250 mL Erlenmeyer flask containing 100 mL of 1.7% potato-dextrose-broth (PDB) medium (1 plug per 100 mL of medium), and incubated at 25°C for 25 days. Therefore, the mycelium was separated from the liquid culture medium by filtration and the secondary metabolism compounds released into the liquid culture medium by the endophytic fungus were extracted by using organic solvents dichloromethane (DCM) and ethyl acetate (EtAc) at 1:2 ratio. After that, all extracts were evaporated in a rotary evaporator under reduced pressure (Rota-evaporador MA120-Marconi) (21).

### Fractionation of crude extracts

Solid phase extraction (SPE) was performed according to Aguiar-Galvão et al. (22), using a Supelclean (C18, 500 mg) reverse phase cartridges. Briefly, 20 mg of sample were dissolved in 200 μL of methanol (MeOH). Cartridge use was preceded by activation of the adsorbent with 5 mL of MeOH, followed by conditioning with 5 mL of milli-Q water. Afterwards, the sample was applied to the cartridge and eluted sequentially with 5 mL of the following eluents: H_2_O (F1); H_2_O/MeOH 25% (F2), H_2_O/MeOH 50% (F3), H_2_O/MeOH 75% (F4), and finally MeOH (F5).This procedure was repeated twice for each sample. Collected fractions were dried in a SpeedVac (Thermo-Fisher) vacuum centrifuge at 40°C for 24 h. Fractions obtained from DCM extract (eDCM) were named as F1_DCM_, F2_DCM_, F3 _DCM_, F4_DCM_, and F5_DCM_, while fractions from EtAc extract (eEtAc) were named as F1_EtAc_, F2_EtAc_, F3_EtAc_, F4_EtAc_, and F5_EtAc_.

#### Ultra-performance liquid chromatography-mass spectrometry (UPLC-MS)

UPLC-MS analysis were performed on a chromatograph coupled to mass spectrometers (UPLC-QTOF Waters Acquity/Xevo) and equipped with an electrospray ionization interface (ESI) and a Waters Acquity BEH C18 column (150 × 2.1 mm, 1.7 μm). Mobile phase was composed by H_2_O (A) and acetonitrile (B), both containing formic acid (0.1% v/v). Elution gradient ranged from 2 to 95%, at a flow rate of 500 μL/min. Samples were pre-filtered on 0.22 μm PTFE syringe filters (Simplepure, United States). The fractions were analyzed in the positive (PI) and negative (NI) ionization modes in a range of 100-1,200 Da. ESI conditions were defined as follows: capillary voltage 2800 V, cone voltage 40 V, source temperature 120°C, dissolution temperature 330°C, cone gas flow of 20 L/h, gas desolvation flow 600 L/h, and MCP (microchannel plate voltage)-detector at 1,900 V. The compound identification was based on the molecular formula deduced from the exact mass (4 decimal places), considering a mass error lower than 5 parts per million (ppm), the isotopic ion pattern (i-fit) and the ion fragmentation pattern compared to literature data (22).

#### Nuclear magnetic resonance spectrometry (NMR)

Hydrogen and Carbon NMR (^1^H and ^13^C), one- and two-dimensional, were accomplished in an DD2Agilent spectrometer (14.1 T), equipped with a 5 mm reverse detection probe, operating at the frequencies of ^1^H and ^13^C at 599.56 and 150.77 MHz, respectively. Samples were dissolved in 0.6 mL of deuterated methanol (MeOD, Cambridge Isotope Laboratories) and analyzed in 5 mm glass tubes. The chemical shifts (δ) were expressed in ppm and referenced by the hydrogen signal of the non-deuterated residual molecules from the deuterated solvent (δH 3.31) and the central carbon peak of the deuterated solvent (δC 49.15). NMR analyses were recorded at 26°C employing basic pulse sequences. For one-dimensional ^1^H and ^13^C experiments the following values were established for the acquisition parameters, respectively: spectral widths of 16 and 252 ppm, acquisition times of 1.7 and 0.865 s, pulse widths of 45° of 4.15 and 3.20 μs (58 dB), number of transients of 16 and 32 K, and relaxation time of 1 s.The one-dimensional experiments were acquired with 32,768 points and processed with 65,356 points. The two-dimensional homonuclear (COSY) and heteronuclear correlation spectra were acquired by pulsed gradient field, employing a number of transients of 16 and 32, respectively. In the COSY, 897 × 128 points were used for the acquisition data matrix and 4,096 × 4,096 points for the processing, while for the HSQC (Heteronuclear Single Quantum Coherence) and HMBC (Heteronuclear Multiple Bond Correlation) experiments 1,142 × 256 points on acquisition and 4,096 × 2,048 points in processing.

### General cell culture procedures

Rat C6, human U87MG and U138MG glioblastoma cell lines were obtained from American Type Cell Collection (Rockville, Maryland, USA). Cells were grown in culture flasks and maintained in Dulbecco's Modified Eagle's Medium (DMEM) (pH 7.4) containing 1% DMEM (Gibco BRL), 8.4 mM HEPES, 23.8 mM NaHCO_3_, 0.1% fungizone, 0.5 U/mL penicillin/streptomycin and supplemented with 10% (v/v) FBS. Cells were kept at 37°C in a humidified atmosphere with 5% CO_2_. Astrocyte cultures were prepared as previously described by Da Frota et al. (23). Briefly, cortex of newborn Wistar rats (1–3 days old) were removed and dissociated mechanically in Ca^+2^ and Mg^+2^-free balanced salt solution (CMF) (pH 7.4) containing 137 mM NaCl, 5.36 mM KCl, 0.27 mM Na_2_HPO_4_, 1.1 mM KH_2_PO_4_, and 6.1 mM glucose. Dissociated tissue was subjected to centrifugation at 1,000 *g* for 5 min. Thereafter, the pellet was suspended in DMEM (pH 7.6) supplemented with 10% FBS. Then, cells (5 × 10^4^) were seeded in poly-L-lysine-coated 96-well plates. Cultures were allowed to grow to confluence by 20–25 days and the medium was replaced every 4 days. All procedures used in the present study followed the “Principles of Laboratory Animal Care” of the National Institutes of Health and were approved by the Ethical Committee of UFPel (CEEA 4755).

### Cell culture treatment

Dried crude organic, fractionated extracts or Sch-642305 were dissolved in DMSO at stock concentration of 10 mg/mL and further diluted in DMEM/10% FBS to obtain a concentration range from 0.625 to 200 μg/mL. Glioblatoma cell lines C6, U87MG and U138MG were seeded at 5 × 10^3^ cells (96-well plates) for cytotoxicity experiments and allowed to grown for 24 h. Astrocyte cultures were prepared as described above. Cell cultures were treated for 24, 48, or 72 h. In order to analyze clonogenic potential of C6 glioma following treatment, cells were seeded in 6-well plates (3 × 10^2^ cells) and treated with crude extracts and fractions at concentrations close to inhibitory concentration 50% (IC_50_) following 48 h of exposure. To perform cell cycle and apoptosis/necrosis analysis, C6 glioma cells were seeded in 6-well plates (1 × 10^5^ cells/well) and treated with eDCM and eEtAc crude extracts and F3_EtAc_ fraction at concentrations close to IC_50_, while compound 1(Sch-642305) was evaluated at 1 μg/mL after 48 h of exposure. In addition, oxidative stress biomarkers were determined in C6 glioma cells seeded in 6-well plates (3 × 10^5^ cells) exposed to eDCM and eEtAc crude extracts, F3_DCM_, F3EtAc and Sch-642305 following 48 h of treatment. Cells exposed to DMSO (0.05% final concentration) were considered control.

### Cytotoxicity study

#### Cell viability assay

Cell viability was evaluated by determination of the soluble 3-(4.5-dimethylthiazol-2-yl)-2.5-diphenyltetrazolium bromide (MTT) reduction by cell dehydrogenases (24). This method is based on the ability of viable cells to reduce MTT and form a blue formazan product. MTT solution (sterile stock solution of 5 mg/mL) was added to the incubation medium in the wells at a final concentration of 0.5 mg/mL. Glioma cells and astrocytes were left for 90 min at 37°C in a humidified 5% CO_2_ atmosphere. The medium was then removed and precipitate was eluted with DMSO. The optical density of each well was measured at 492 nm in a microplate reader (SpectraMAX 190). Results were expressed as percentage of control.

#### Cell proliferation assay

Sulforhodamine B (SRB) colorimetric assay was used for cell density and cytotoxicity determination, based on staining of total cell protein content with SRB dye (25). Briefly, cultures were washed and fixed with 50% trichloroacetic acid (w/v) for 30 min (4°C); cells were washed 5 times with dH_2_O, stained with 0.4% SRB (w/v) for 45 min (RT) and washed 5 times with 1% acetic acid (v/v). Finally, SRB complexes were eluded in 10 mM Tris buffer following by 15 min shaking. Absorbance was measured at 540 nm in a microplate reader (SpectraMAX 190). Results were expressed as percentage of control.

#### Cell migration assay

In order to investigate changes in cell migration, the wound healing assay was carried out as described in a previous report (26). Briefly, a pipette-200 tip was used to create a lesion in the cell monolayer to generate the “wound.” C6 and U138MG cell cultures were washed with PBS to remove debris and cells were treated with Sch-642305 (0.5 μg/mL). Closure of the wound was monitored in an inverted microscope (40x) at time intervals of 0, 18, 24, and 48 h after scratching the monolayer. Quantitative analysis of the cell-free slit was measured by software ImageJ 1.51j8 (National Institutes of Health, USA) and the inhibition of cells migration was expressed in percentage.

#### Clonogenic assay

Clonogenic assay is an *in vitro* cell survival method based on the capability of a single cell to grow into a colony, which can be used to determine the effectiveness of cytotoxic agents (27). Following 48 h treatment, C6 cells (3 × 10^2^ cell/well) were seeded in 6-well plates and cultured for additional 10 days in absence of treatment. Then, cells were fixed with ice-cold methanol (100%) and stained with crystal violet 1% (w/v) to visualize colonies. Colonies were counted using microscope (40x) and length of colonies were determined using software ImageJ 1.51j8 (National Institutes of Health, USA).

#### Cell cycle analyses

Following 48 h of treatment, cell cycle analyses was performed as described by Viau et al. (28). The medium and the cells were harvested and centrifuged (10 min; 1,000 *g*). Supernatant was removed and cell pellet was washed once with PBS and fixed with 70% EtOH. After 2 h, cells were washed and incubated with staining solution (1% Triton X-100, 2 mg/mL RNase, 2 mg/mL propidium iodide (PI) in PBS). After 30 min, data were collected using FACS Calibur Flow Cytometer (BD Bioscience, Mountain View, CA, United States). Results were expressed as percentage of control.

#### Cell death analyses

Apoptotic or necrotic cells were quantified using annexinV-FITC-PI double staining as described by Viau et al. (28). Following 48 h of treatment, the medium and the cells were harvested and centrifuged (10 min at 2,000 *g*). Cell pellet was washed twice with PBS and it was incubated (5 min, RT) with a biding buffer containing FITC-conjugated annexin V and PI, following manufacture instructions. Apoptotic and/or necrotic cells were quantified using FACS Calibur Flow Cytometer (BD Bioscience, Mountain View, CA, USA). Cells were classified as follows: viable cells (Annexin^−^/PI^−^), early apoptotic (Annexin^+^/PI^−^), late apoptotic (Annexin^+^/PI^+^) or necrotic cells (Annexin^−^/PI^+^). Results were expressed as percentage of control.

### Oxidative stress parameters

#### Determination of reactive oxygen species (ROS)

Intracellular generation of ROS was determined by DCFH assay as described by Dos Santos et al. (29). This method is based on the oxidation of 2′-7′- dichlorodihydrofluorescein diacetate (DCFH-DA) to dichlorofluorescin (DCFH) by intracellular esterases, which is trapped within the cell. Thereby, DCF-DA reacts with ROS emitting fluorescence. In brief, following 48 h of treatment, cultures were incubated with 1 μM DCFH-DA for 30 min and fluorescence was measured at 488/525 nm in a microplate reader (SpectraMax M3). ROS production was reported as percentage of control.

#### Superoxide dismutase (SOD) activity

SOD activity was assayed as described by Misra and Fridovich (30). This assay is based on the inhibition of superoxide-dependent adrenaline auto-oxidation to adenochrome. This reaction is intermediate by superoxide, which is scavenged by SOD. The absorbance was measured at 480 nm in a microplate reader (SpectraMax M3) and the results were expressed as percentage of control.

#### Catalase (CAT) activity

CAT activity was measured as described by Aebi (31). This assay is based on the decomposition of 30 mM hydrogen peroxide (H_2_O_2_) in 50 mM potassium phosphate buffer (pH 7.0) continuously monitored at 240 nm for 180 s at 37°C. CAT activity was reported as percentage of control.

#### Glutathione peroxidase (GPx) activity

GPx activity was measured using a commercial kit (RANSEL®; Randox Lab, Antrim, United Kingdom). This assay is based on oxidation of GPx catalyses glutathiose (GSH) by cumene hydroperoxide. In presence of glutathione reductase (GR) and NADPH, the oxidized glutathione (GSSG) is immediately converted to the reduced form with concomitant oxidation of NADPH to NADP^+^. NADPH disappearance was measured at 340 nm and GPx activity was reported as percentage of control.

#### Total sulfhydryl content quantification

Total sulfhydryl content was determined according to Aksenov and Markesbery (32). This process is based on the reduction of 5,5'-dithio-bis(2-nitrobenzoic acid) (DTNB) by thiols, whose reaction form an oxidized disulfide generating a yellow derivative (TNB). The reaction was started by the addition of 5,5′-dithio-bis(2-nitrobenzoic acid) (DTNB).The absorbance was measured at 412 nm in a microplate reader (SpectraMax M3) and the results were expressed as percentage of control.

### Statistical analysis

Statistical analysis was carried using GraphPad Prism 5 software. Data were expressed as mean ± standard error (SEM) and were subjected to analysis of variance (ANOVA) followed by Tukey *post-hoc* test for multiple comparisons. Differences between mean values were considered significant when *P* < 0.05.

## Results

### Isolation and identification of endophytic fungus

Here, endophytic fungus was isolated from stems of *A. satureioides* and named as MF31b11. Endophyte exhibited filamentous colonies with cottony aspect and regular edge, which color ranges from white to brown. Upon microculture analysis, septate conidiophores and cylindrical phialides were observed at microscope. However, structural characterization of the conidia and phialides difficulties did not allow morphological identification of the endophyte.

### Endophytic fungus extracts selectively decrease glioma cell proliferation and viability

**Figure 1:**
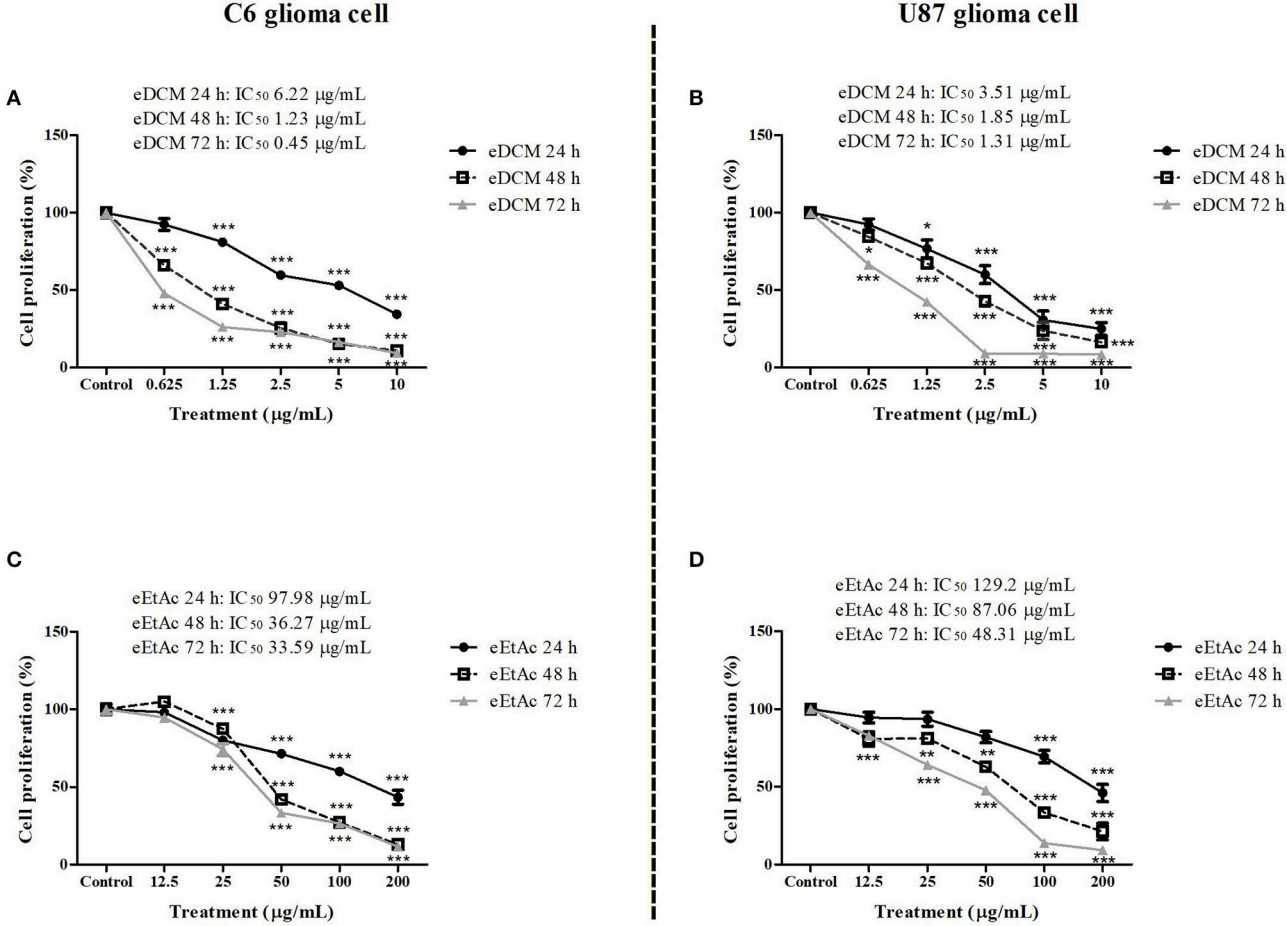
Comparative effect of eDCM and eEtAc on glioma cell proliferation. C6 (left panel) and U87MG (right panel) glioma cell lines were exposed to increasing concentrations of eDCM **(A,B)** and eEtAc **(C,D)**. Cell proliferation was determined by SRB assay following 24, 48, or 72 h of treatment, as indicated. Values represent the mean ± SEM from at least three independent experiments performed in triplicate. Data were analyzed by ANOVA followed by *post-hoc* comparisons (Tukey test). *, **, *** Significantly different from control cells (*P* < 0.05, *P* < 0.01, and *P* < 0.001, respectively).

**Figure 2:**
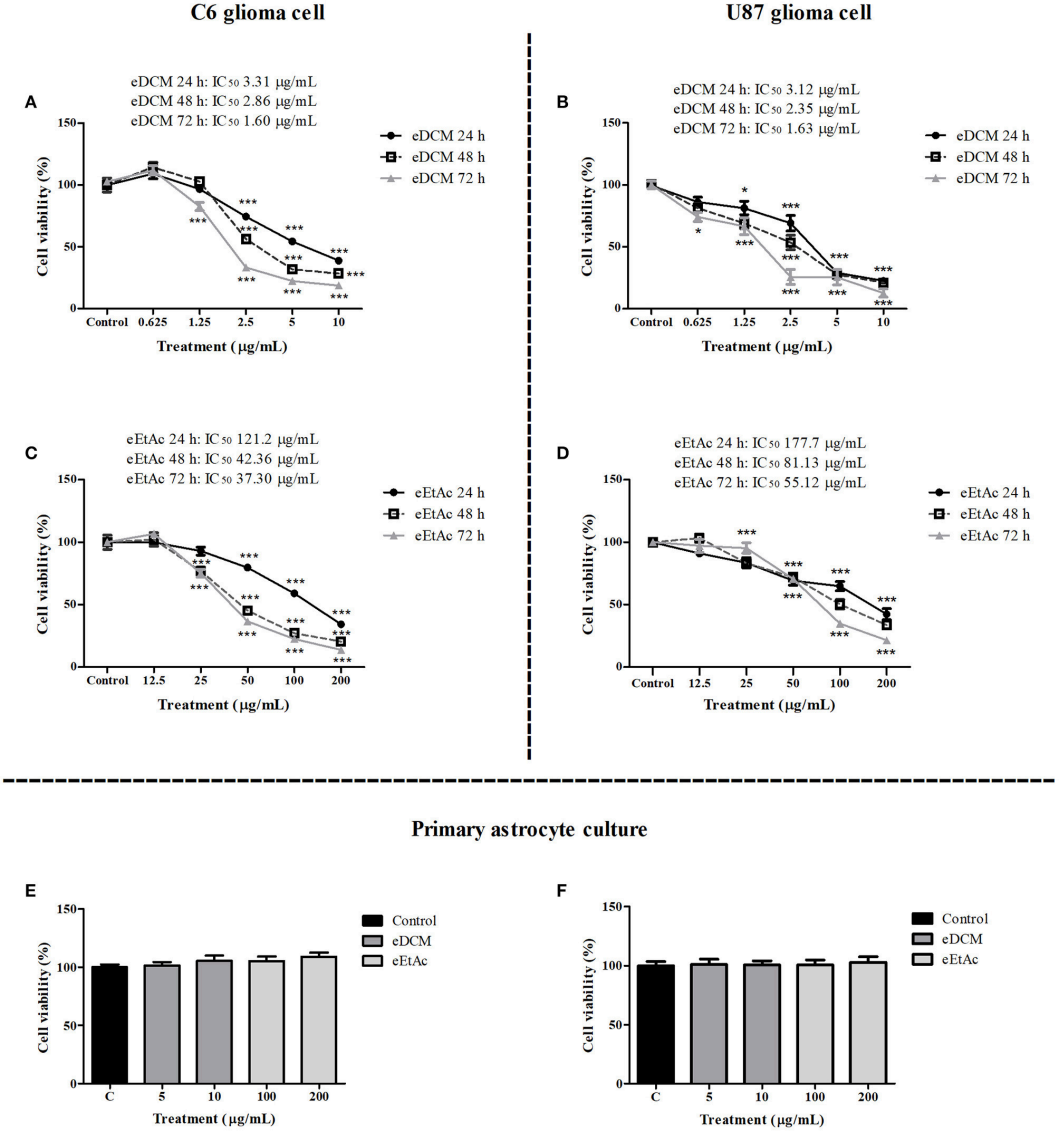
Comparative effect of eDCM and eEtAc on glioma cell viability. C6 (left panel) and U87MG (right panel) glioma cell lines were exposed to increasing concentrations of eDCM **(A,B)** and eEtAc **(C,D)** for 24, 48, or 72 h of treatment, as indicated. In addition, primary astrocyte cultures were exposed to higher concentrations of crude extracts for 48 h **(E)** and 72 h **(F)**. Cell viability was determined by MTT assay. The values represent the mean ± SEM from at least three independent experiments performed in triplicate. Data were analyzed by ANOVA followed by *post-hoc* comparisons (Tukey test). *, *** Significantly different from control cells (*P* < 0.05 and *P* < 0.001, respectively).

In order to evaluate whether MF31b11 exhibits antitumor activity, in the first set of experiments the liquid culture of isolated fungus was submitted to extraction with DCM and EtAc, which were chosen to isolate molecules with differential chemical properties. The resultant crude extracts (eDCM and eEtAc) were used to determine the citotoxicity of secondary metabolites produced by MF31b11. Rat C6 and human U87MG glioma cell lines were exposed to increasing concentrations of crude extracts for 24, 48, or 72 h. Extract concentrations applied were determined in previous experiments (data not shown) and ranged from 0.625–10 μg/mL to 12.5–200 μg/mL for eDCM and eEtAc, respectively. As shown in Figure 1, eDCM and eEtAc inhibited ~90% C6 (Figures 1A,C) and U87MG (Figures 1B,D) glioma cell proliferation following 72 h of treatment and the effect was time and concentration-dependent, as determined by SRB assay. Additionally, eDCM exhibited stronger antitumor activity when compared to eEtAc in both glioma cell lines as indicated by IC_50_ values, which ranged from 0.45–6.22 μg/mL to 33.59–129.2 μg/mL for eDCM and eEtAc, respectively (Figure 1). The effect of crude extracts on glioblastoma cell viability was also evaluated by MTT assay (Figure 2). Results show that both extracts decreased glioma viability in a time-dependent manner and eDCM was more cytotoxic than eEtAc (Figures 2A–D), confirming the previous data (Figure 1). Notably, eDCM and eEtAc did not alter normal astrocyte cell culture viability (Figures 2E,F), indicating selective effect against tumor cells.

### Fractionated extracts exhibit selective antiglioma effect

**Figure 3:**
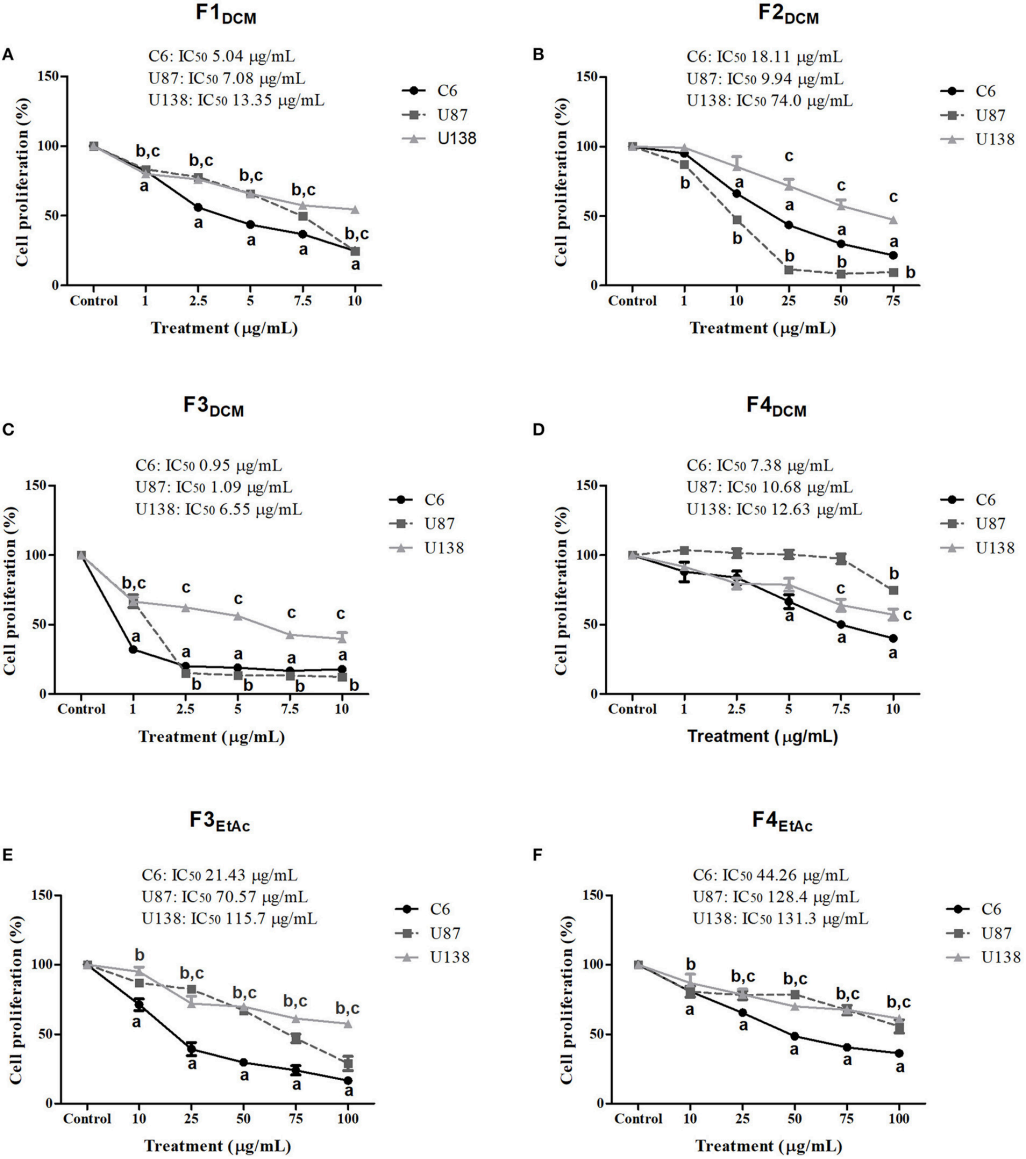
Comparative effect of fractionated extracts from eDCM and eEtAc on glioma cell proliferation. Glioma cell lines were exposed to increasing concentrations of F1_DCM_, F2_DCM_, F3_DCM_, F4_DCM_, F3_EtAc_, and F4_EtAc_ fractions (**A–F**, respectively). Cell proliferation was determined by SRB assay following 48 h of treatment. The values represent the mean ± SEM from at least three independent experiments performed in triplicate. Data were analyzed by ANOVA followed by *post-hoc* comparisons (Tukey test). ^a, b, c^ Significantly different from C6, U87MG, and U138MG control cells, respectively (*P* < 0.001).

**Figure 4:**
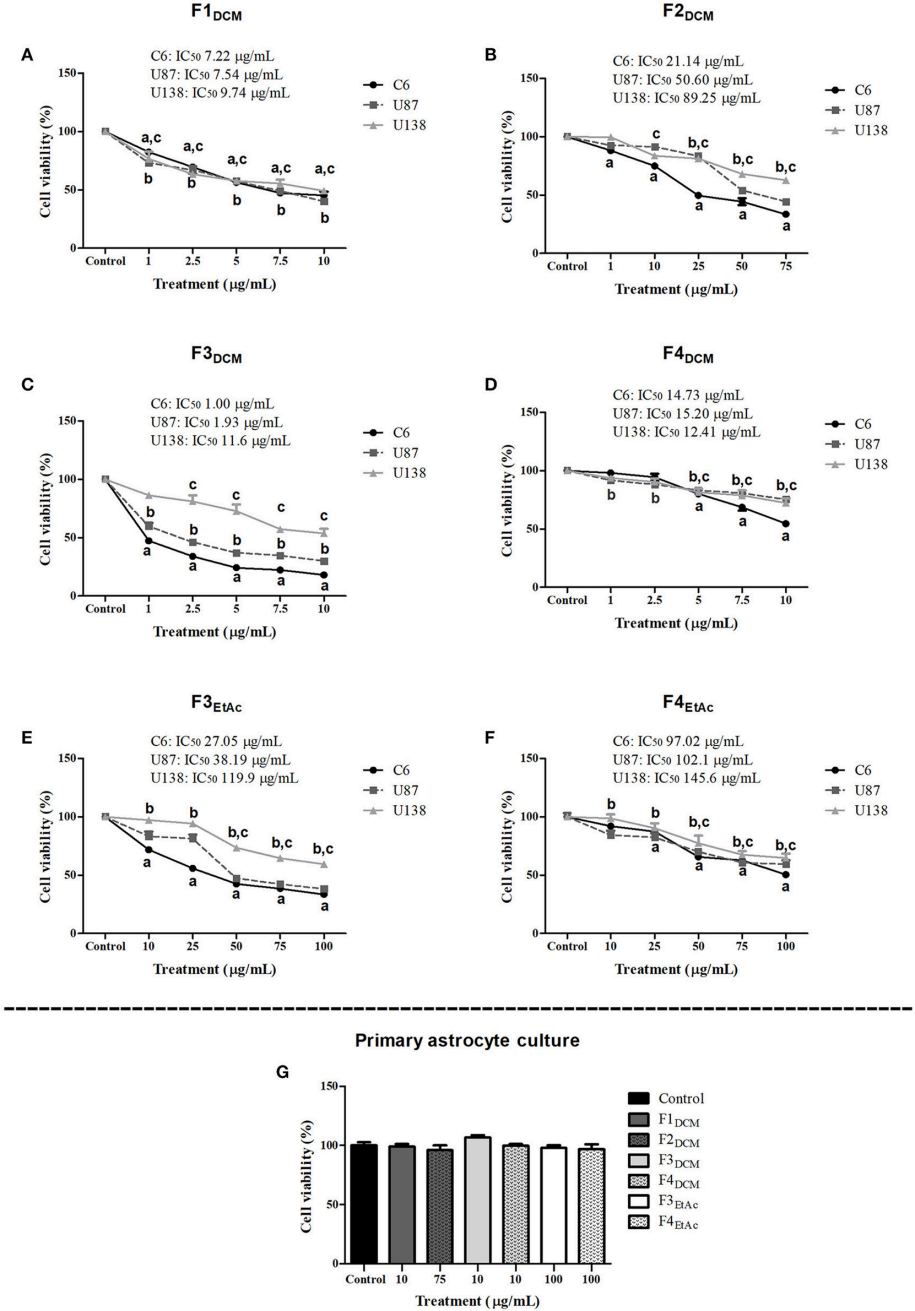
Comparative effect of fractionated extracts from eDCM and eEtAc on glioma and astrocyte cell viability. Glioma cell lines were exposed to increasing concentrations of F1_DCM_, F2_DCM_, F3_DCM_, F4_DCM_, F3_EtAc_and F4_EtAc_ fractions (**A–F**, respectively). In addition, primary astrocyte cultures **(G)** were exposed to the higher concentration of F1_DCM_ (10 μg/mL), F2_DCM_ (75 μg/mL), F3_DCM_ (10 μg/mL), F4_DCM_ (10 μg/mL), F3_EtAc_ (100 μg/mL) and F4_EtAc_ (100 μg/mL) fractions. Cell viability was determined by MTT test after 48 h of treatment. The values represent the mean ± SEM from at least three independent experiments performed in triplicate. Data were analyzed by ANOVA followed by *post-hoc* comparisons (Tukey test). ^a, b, c^ Significantly different from C6, U87MG, and U138MG control cells, respectively (*P* < 0.001).

To better investigate which molecule(s) were involved in eDCM and eEtAc antiglioma activity, crude extracts were fractioned as described in material and methods and its citotoxicity was determined in C6, U87MG, and U138MG glioma cells by SRB and MTT assays as above. From 10 obtained fractions, 6 identified as F1_DCM_, F2_DCM_, F3_DCM_, F4_DCM_, F3_EtAc_, and F4_EtAc_ showed significant cytotoxic activity against glioma cell lines (Figure 3). The variability of antitumor effect could be observed by IC_50_ values, which ranged from 0.95 to 131.3 μg/mL and may be related to differential chemical properties exhibited by these fractions, as well as the cell line tested, as expected. Citotoxicity analyses were also performed using MTT assay at the same experimental conditions (Figure 4). In a general way, cell viability results and IC_50_ values obtained were according to antitumor potential exhibited by these fractions. Interestingly, although the antiglioma effect exerted by fractions, no alteration was observed in primary astrocyte cell viability after 48 h of exposure (Figure 4G), reinforcing selective antiglioma activity.

### Extracts and fractions of endophytic fungus decrease glioma cell colony formation

**Figure 5:**
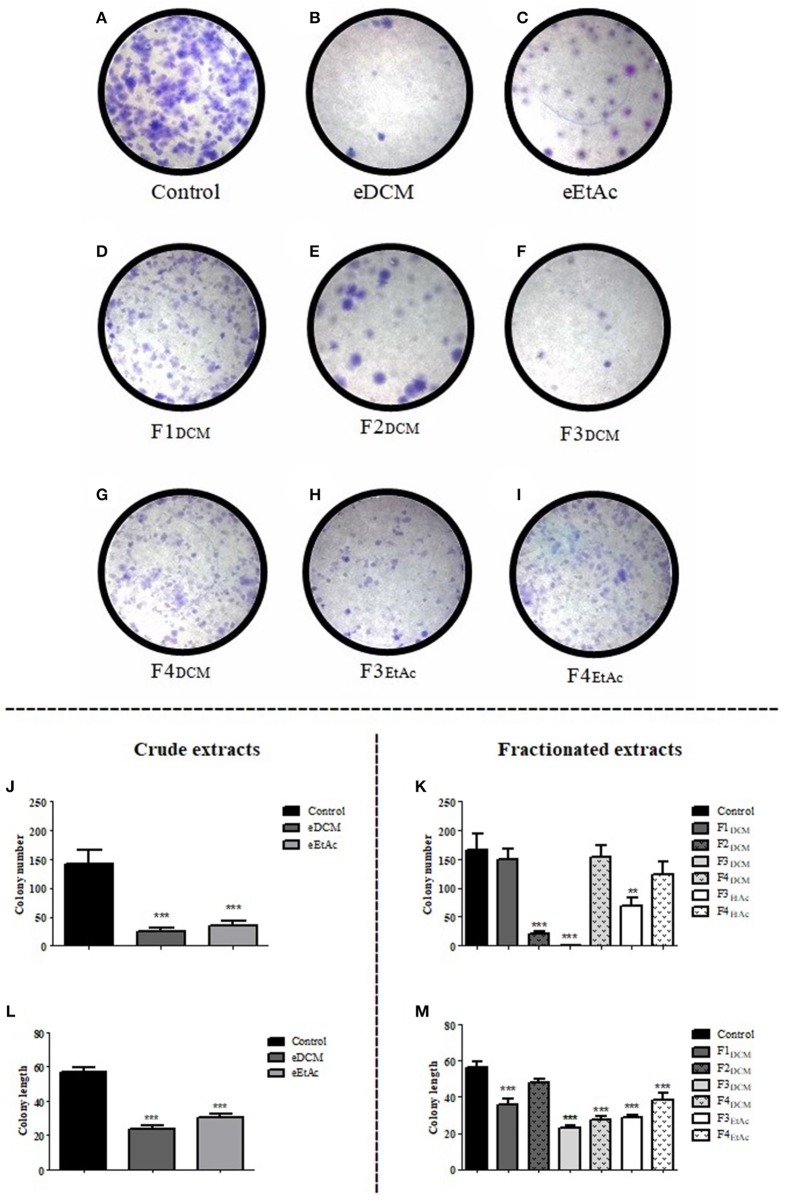
Analysis of crude organic and fractionated extracts effect on C6 glioma colony formation. Glioma cells were exposed to eDCM (2.5 μg/mL), eEtAc (50 μg/mL) crude extracts and F1_DCM_ (7.5 μg/mL), F2_DCM_ (25 μg/mL), F3_DCM_ (1 μg/mL), F4_DCM_ (10 μg/mL), F3_EtAc_ (25 μg/mL), and F4_EtAc_ (100 μg/mL) fractions at concentrations close to IC_50_ value **(A–I)**. Colony number **(J,K)** and colony length **(L,M)** were determined by clonogenic assay following 48 h of treatment. Values represent the mean ± SEM from at least three independent experiments performed in triplicate. Data were analyzed by ANOVA followed by *post-hoc* comparisons (Tukey test). ^*^, ^**^, ^***^ Significant different from control cells (*P* < 0.01 and *P* < 0.001 respectively).

Clonogenic assay was employed to determine the reproductive cell death and effectiveness of cytotoxic agents (27). C6 cells were treated with crude extract or fractions at concentration corresponding to IC_50_ and colony length and formation was determined as described in material and methods. eDCM and eEtAc crude extracts, F2_DCM_, F3_DCM_, and F3_EtAc_ fractions decreased in 83, 76, 88, 99, and 58%, respectively, C6 glioma colony formation when compared to control (Figures 5A–K). Colony length was also decreased by 59, 46, 37, 59, 52, 49, and 33% following exposure to eDCM, eEtAc, F1_DCM_, F3_DCM_, F4_DCM_, F3_EtAc_, and F4_EtAc_, respectively (Figures 5L,M). Taken together, data obtained from clonogenic, proliferation and cell viability analyses point F3_DCM_ as the most effective antiglioma fraction when compared to the others. Therefore, further experiments of phytochemical analysis of this fraction were performed.

### Structural elucidation of compound 1

^1^H-NMR spectrum (MeOD) of the metabolite isolated from F3_DCM_ (Table 1) exhibited signals at δ 7.93 (1H, d, H-3) and 5.96 (1H, dd, *J* = 10 Hz, H-2), which were characteristics of oleofinic hydrogens. Signals at δ 5.05 (1H, m, H-11) and 4.22 (1H, t, *J* = Hz, H-4) were related to hydrogens of oxygenated carbon. Moreover, eight signals in the region of δ 2.83 −1.28 were attributed to alkyl hydrogens. The ^13^C NMR spectrum (Table 1) showed fourteen resonances, of which one was consistent with ketone carbonyl (δ 202.5, C-1) and one with ester carbonyl (δ 173.8, C-12). Resonances at δ 149.5 (C-12) and δ 130.7 (C-2) were associated to olefinic carbons, while signals at δ 74.8 (C-11) and δ 67.2 (C-4) were attributed to oxygenated sp^3^ carbons. The eight remaining signals were associated to alkyl carbons (Table 1).

**Table 1:** ^1^H and ^13^C NMR data (one and two dimensional) of Sch-642305 (MeOD).

**#C**	**δC 1**	**δH 1**	**HMBC**	**COSY**
C = O	202.5		7.03; 2.66
C = O	173.8		2.68; 2.54
=CH	149.5	7.03 (1H.dd)	4.22	5.96; 4.22
=CH	130.7	5.96 (1H.d. 10Hz)	4.22	7.03
CH	74.8	5.05 (1H.m)	2.54; 1.84	1.28
CH	67.2	4.22 (t	7.03; 5.96; 2.54; 1.28; 1.40	7.03
CH	47.8	2.66	5.96; 4.22; 2.68; 2.54; 2.17; 1.09
CH_2_	39.9	2.68; 2.54	2.83	2.83
CH	37.9	2.83 (t)	7.03; 2.68; 2.54; 2.17	2.68; 2.54
CH_2_	30.5	1.40 (m)	1.28	2.07
CH_2_	24.3	2.17 (m); 1.09 (t)	2.66; 1.40; 1.09	2.16; 1.56
CH_2_	24.2	1.84; 1.24	5.05	1.84; 1.24
CH_2_	22.8	1.56; 1.34	2.66; 1.84; 1.40; 1.09	1.09
CH_3_	18.7	1.28 (6H.d)	

The edited ^1^H-^13^C HSQC-NMR of compound **1** revealed the correlations of the hydrogenated carbons and allowed differentiating the CH and CH_3_ signals from CH_2_. Thus, 6 methylene carbons, 5 methylene carbons, 1 methyl carbon and 2 non-hydrogenated carbons (carbonyls) were identified. The couplings observed in the COSY spectrum (Table 1) confirmed the vicinal and geminal hydrogens of the alicyclic carbon chain.

**Figure 6:**
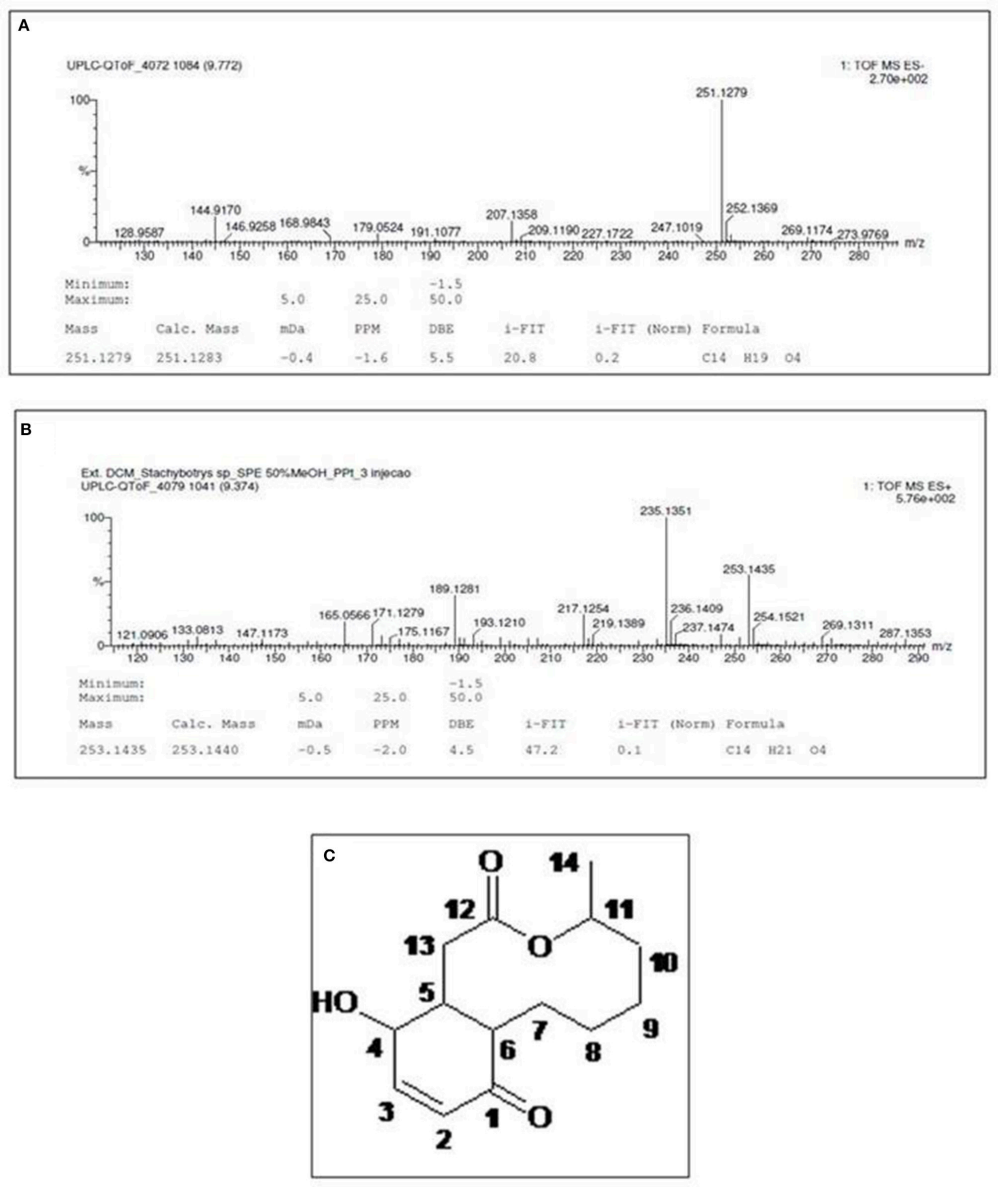
High-resolution mass spectrum of compound 1, obtained in negative **(A)** and positive **(B)** ionization modes. **(C)** Structure of characterized molecule Sch-642305.

The high resolution mass spectrum (Figure 6) exhibited precursor ions [M+H]^+^ at *m/z* 251.1885 and [M-H]^−^at*m/z* 253.1435 consistent with the molecular formula C_14_H_20_O_14_, suggesting a bicyclic hydroquinone lactone.

^1^H-^13^C HMBC-NMR spectrum (Table 1) allowed the characterization of the hydroquinone ring through the following correlations to two and three bonds: olefinic hydrogens at δ_H_ 7.03 and 5.96 (H-3 and 2) with the carbonyl at δ_C_ 200.3 (C-1) and the carbinolic carbon at δ_C_ 67.2 (C-4). Lactone was characterized by the coupling of aliphatic hydrogen at δ_H_ 2.68 (H-11) and hydrogen bonded to oxygenated carbon at δ_H_ 5.05 (H-11) with the carbonylat δ_C_ 173.8 (C-12). The hydroquinone and lactone ring junctions were confirmed by the coupling of the methinic hydrogens at δ_H_ 2.83 (H-5) and 2.66 (H-6) with the methilene carbons at δ_C_ 39.9 (C-13) and 24.3 (C-7), respectively, besides the ketone carbonyl at δ_C_ 200.3 (C-1). Finally, comparison of ^1^H and ^13^C NMR data with those in the literature confirmed the chemical structure of compound 1 (Figure 6C) (33), which was characterized as (4S,8aR,12S,12aR)-12-hydroxy-4-methyl-4,5,6,7,8,8a,12,12a-octahydro-1H-3-benzoxecine-2,9-dione (IUPAC name), a 10-membered macrolide (macrocyclic lactone) known as Sch-642305.

### Sch-642305 promoted antiproliferative effect, decreased glioma cell colony formation and migration

**Figure 7:**
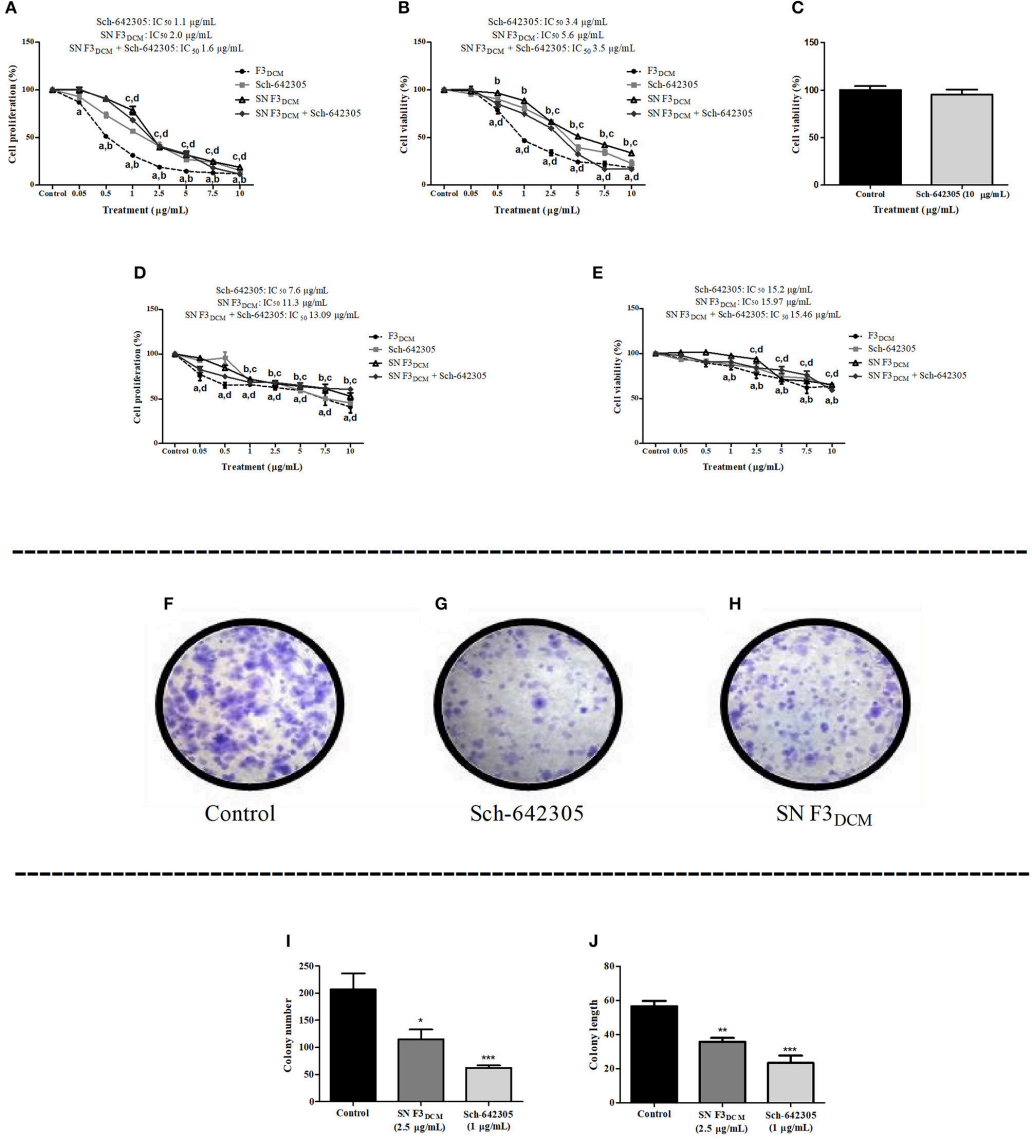
Analysis of Sch-642305, SN F3_DCM_ and association SN F3_DCM_+Sch-642305 effect cytotoxicity (C6, U138MG and astrocytes) and colony formation (C6 glioma). Cytotoxic activity on C6 and U138MG glioma cells was determined by SRB **(A,D)** and MTT assays **(B,E)**. Cell viability of primary astrocyte culture was measured by MTT **(C)**. Colony number and colony length were determined in C6 cells by clonogenic assay following 48 h of treatment **(F–J)**. Values represent the mean ± SEM from at least three independent experiments performed in triplicate. Data were analyzed by ANOVA followed by *post-hoc* comparisons (Tukey test). *, **, ***Significantly different from control cells (*P* < 0.05, *P* < 0.01 and *P* < 0.001, respectively). ^a, b, c, d^ Significantly different from F3_DCM_, Sch-642305, SN F3_DCM_ and SN F3_DCM_+Sch-642305 control cells, respectively (*P* < 0.001).

Sch-642305 induced antiproliferative activity against C6 and U138MG glioma cells, exhibiting a concentration-dependent profile for both cell lines evaluated following 48 h of treatment. The IC_50_ values obtained for C6 cells was 1.1 and 3.4 μg/mL (4.36 and 13.5 μM) and for U138MG was 7.6 and 15.20 μg/mL(30.1 and 60.24 μM) for SRB and MTT assays, respectively, as indicated in (Figures 7A,B,D,E). Interestingly, Sch-642305 did not alter primary astrocyte cell viability (Figure 7C), suggesting a selective effect against glioma cells. The cytotoxic activity of F3_DCM_ fraction without the presence of lactone, named as “supernatant F3_DCM_” (SN F3_DCM_) was also investigated. SN F3_DCM_ exhibited antiproliferative effect against C6 and U138MG glioma cells. The IC_50_ values obtained for C6 cells was 2.0 and 5.6 μg/mL and for U138MG was 11.3 and 15.97 μg/mL for SRB and MTT assays, respectively (Figures 7A,B,D,E). The exposure of C6 or U138MG glioma cells to Sch-642305 and SN F3_DCM_ in association induced cytotoxicity with values of IC_50_ 1.6/13.09 and 3.5/15.43 μg/mL as determined by SRB and MTT assays, respectively (Figures 7A,B,D,E).

**Figure 8:**
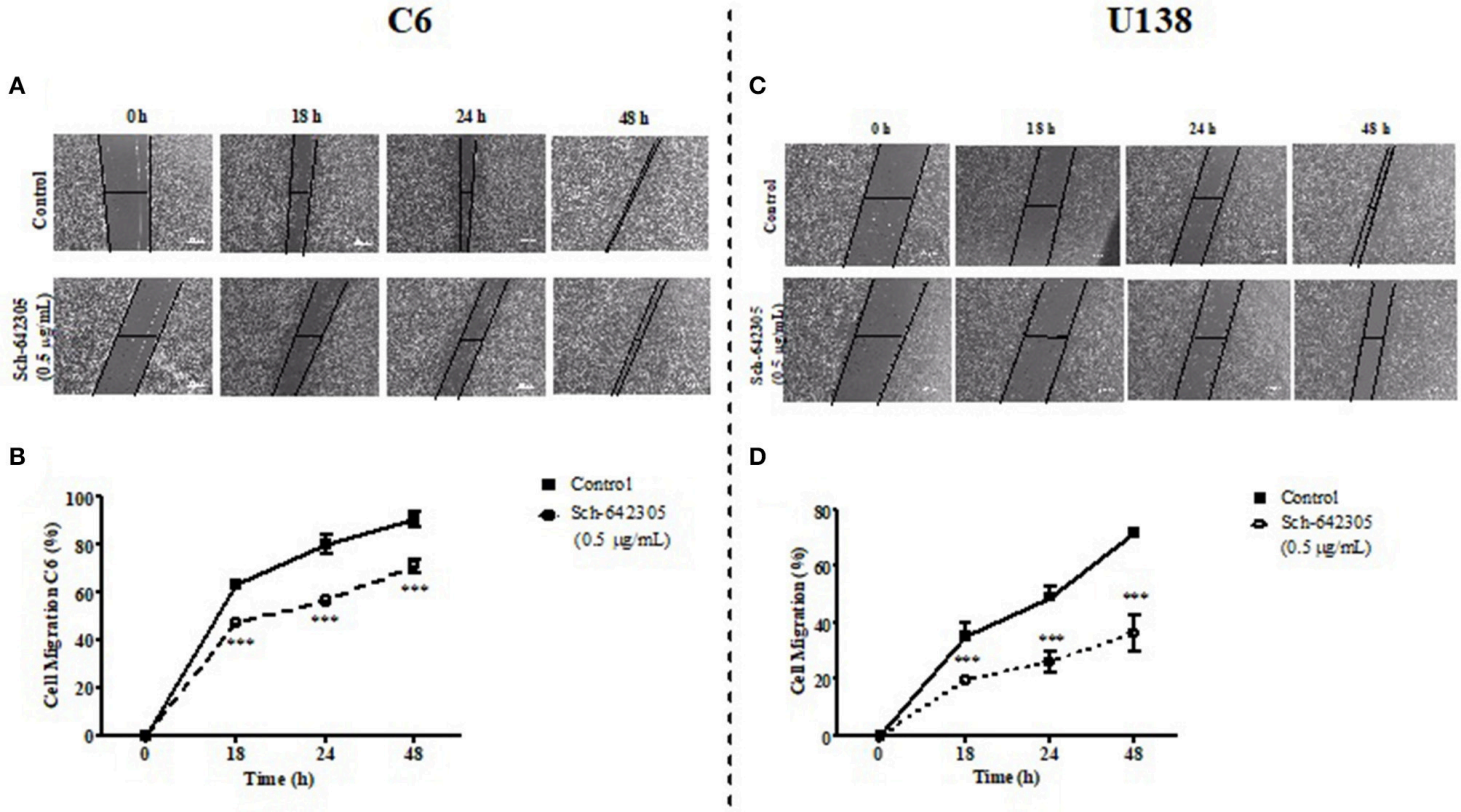
Analysis of Sch-642305 effect on C6 and U138MG glioma cell migration. C6 **(A,B)** and U138MG **(C,D)** glioma cells exposed to Sch-642305 (0.05 μg/mL). Cells were treated for 48 h and subsequently analyzed by capturing images in an inverted microscope (40x) at time intervals of 0, 18, 24, and 48 h after scratching the monolayer. The percentage of cell migration was determined by software ImageJ and GraphPad Prism. Data were analyzed by ANOVA followed by *post-hoc* comparisons (Tukey test). ***Significant different from control cells (*P* < 0.001).

The potential of Sch-642305 and SN F3_DCM_ to modulate glioma clonogenic activity was determined in C6 cells. Sch-642305 (1.1 μg/mL or 4.36 μM) or SN F3_DCM_ (1 μg/mL) decreased C6 glioma colony formation by 70 and 45%, respectively, and reduced the colony length by 58 and 37%, respectively, when compared to control (Figures 7F–J). Finally, Sch-642305 (0.5 μg/mL or 1.98 μM) inhibited 20 and 36% C6 and U138MG cell migration, respectively, when compared to control cells, following 48 h of exposure (Figures 8A–D). These results indicate that Sch-642305 contributed significantly to antitumor activity exhibited by F3_DCM_ fraction. Therefore, it becomes interesting to elucidate the mechanisms involved in this effect.

### The effect of extracts and fractions of endophytic fungus on cell cycle distribution and cell death

**Figure 9:**
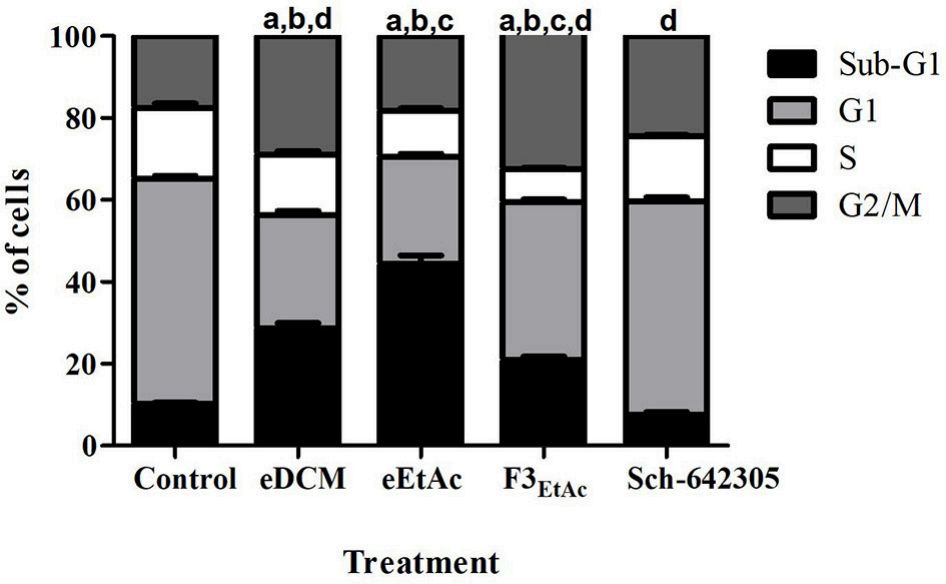
Analysis of crude extracts, fractions and Sch-642305 effect on C6 glioma cell cycle. C6 glioma cells exposed to eDCM (1 μg/mL), eEtAc (50 μg/mL), F3_EtAc_ (25 μg/mL) and Sch-642305 (1 μg/mL). Cells were treated for 48 h and subsequently analyzed by flow cytometry using PI staining to determine the distribution of cells on cell cycle. The percentage of cells on sub-G1, G1, S, and G2/M phases were shown in the graph.^a, b, c, d^ Significantly different from Sub-G1, G1, S, and G2/M phases of control cells, respectively (*P* < 0.001).

**Figure 10:**
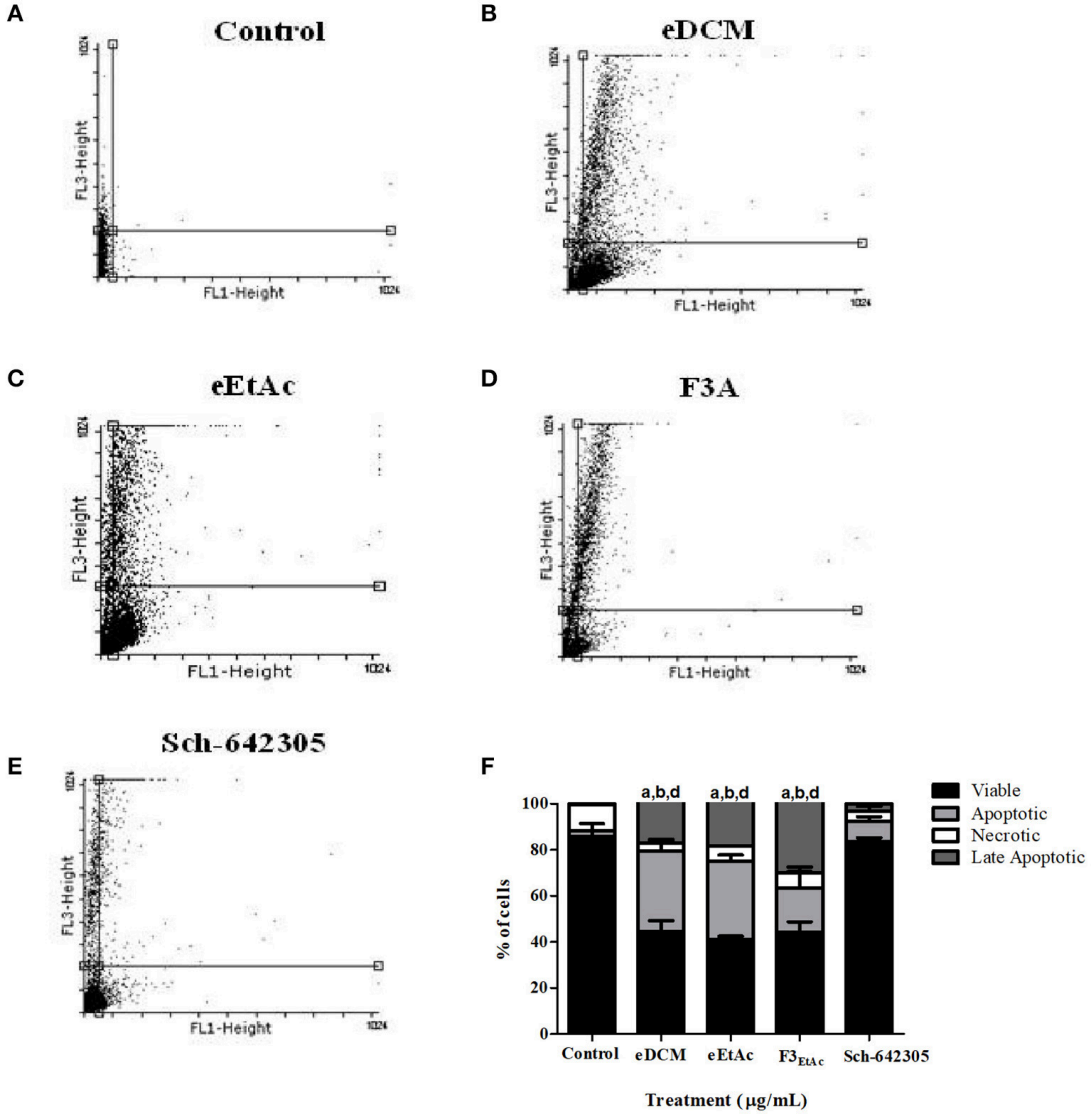
Analysis of crude extracts, fractions and Sch-642305 effect on C6 glioma cell death. C6 glioma was exposed to eDCM (1 μg/mL), eEtAc (50 μg/mL), F3_EtAc_ (25 μg/mL) and Sch-642305 (1 μg/mL). Cells were treated for 48 h and subsequently analyzed by flow cytometry using annexin V/PI double staining to determine cell death by apoptosis, late apoptosis or necrosis. **(A–E)**. The percentage of viable cells is represented by the lower left quadrant; the percentage of apoptosis is represented by the lower right, the percentage of late apoptosis is represented by the upper right and the percentage of necrosis is represented by the upper left; **(F)** representative graph of cell death analysis. ^a, b, d^Significantly different from viable, apoptotic, and late apoptotic of control cells, respectively (*P* < 0.001).

To better understand the antiproliferative effect mediated by crude extracts as well as the most effective fraction of eEtAc (F3_EtAc_) and Sch-642305 compound from F3_DCM_, cell cycle and cell death analyses were performed in C6 glioma following 48 h of treatment. Analysis of cell cycle distribution evidenced that eDCM (2.5 μg/mL), eEtAc (50 μg/mL) and F3_EtAc_ (25 μg/mL) lead G2/M phase arrest of the cell cycle (11.34, 0.65, and 14.88%, respectively) and the formation of sub-G1 apoptotic cells (18.33, 34.08, and 10.61%, respectively; Figure 9),which are in accordance to apoptosis and late apoptosis rates observed in these cells by annexin V-PI staining (33/17%; 32/18%; 17/30%, respectively; Figures 10A–E: dot-plot data; panel F: quantification of annexin-V and/or PI cell staining). The isolated molecule, Sch-642305 (1 μg/mL) induced a cell blockage in G2/M phase (7%; Figure 9) and ~10% of apoptosis/late apoptosis in C6 cells (Figures 10E,F). These results suggest that the induction of C6 cell cycle blockage and apoptosis by extracts, fractions and Sch-642305 account at least in part for its antiglioma activity.

### Extracts and fractions of endophytic fungus alters oxidative stress parameters in glioma cells

**Figure 11:**
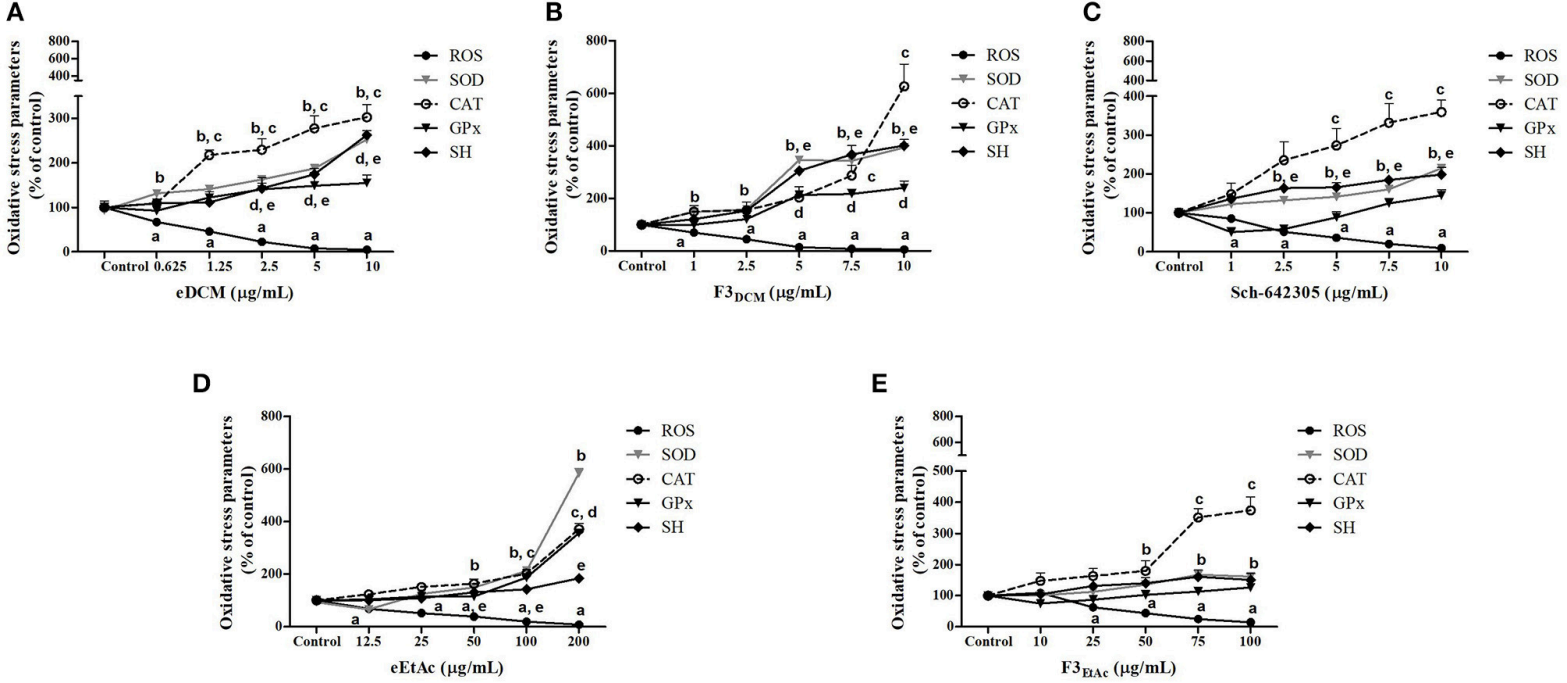
Analysis of oxidative stress parameters in C6 glioma cells exposed to extracts and Sch-642305 macrolide of endophytic fungi from *A. satureioides* for 48 h. **(A)** eDCM extract; **(B)** F3_DCM_ extract; **(C)** Sch-642305; **(D)** eEtAc extract and **(E)** F3_EtAc_ extract. Values represent mean ± SEM of at least three independent experiments. Data were analyzed by ANOVA followed by *post-hoc* comparisons (Tukey test). ^a, b, c, d, e^Significantly different from control cells of ROS, SOD, CAT, GPx, and SH oxidative parameters, respectively.

**Figure 12:**
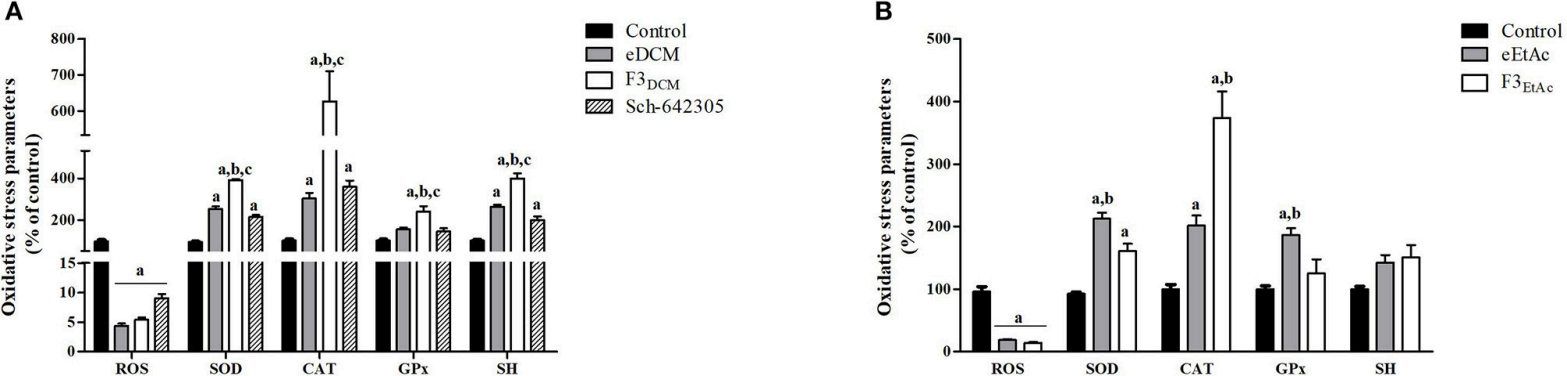
Comparative analysis of oxidative stress parameters in C6 glioma cells exposed to **(A)** eDCM, F3_DCM_, or Sch-642305 (10 μM); **(B)** eEtAc or F3_EtAc_ (100 μM). Analyses were performed after 48 h of treatment. Values represent mean ± SEM of at least three independent experiments. Data were analyzed by ANOVA followed by *post-hoc* comparisons (Tukey test). ^a, b, c^Significantly different from control cells, crude extract (eDCM or eEtAc) and Sch-642305, respectively (*P* < 0.05).

Alterations in ROS parameters are related to cancer progression (6). Indeed, C6 glioma cells exhibit increased ROS levels (5.7 fold; 752.56 *vs*. 191.95 nmol DCF/mg of protein) and SOD activity (1.98 fold; 479.41 *vs*. 338.45 U/mg of protein), while CAT activity was decreased (3.4 fold; 5.05 *vs*. 16.61 U/mg of protein) when compared to primary astrocyte cultures. Therefore, hydrogen peroxide accumulation due to increased SOD and reduced CAT activities may contribute to tumor cell proliferation. In line with this, redox potential of eDCM and eEtAc crude extracts and its respective more effective fractions was further evaluated in glioma cells. Notably, crude extracts increased in a concentration-dependent fashion antioxidant enzyme activities, namely SOD, CAT and GPx by 155, 200, and 55%, respectively, after treatment with eDCM (10 μg/mL; Figure 11A) and by 485, 270, and 255%, respectively, following treatment with eEtAc (200 μg/mL) when compared to control (Figure 11D). Additionally, eDCM and eEtAc induced an increase in total sulfhydryl (SH) content by 160% (10 μg/mL) and 80% (200 μg/mL), respectively (Figures 11A,D). In accordance to antioxidant activity of both extracts, ROS content was also decreased in a concentration-dependent manner when compared to control (Figures 11A,D). F3_DCM_ fraction accounts for significant antioxidant activity exhibited by eDCM crude extract. As shown in (Figure 11B), F3_DCM_ increased by 300, 500, 240 and 300% SOD, CAT, Gpx activities and SH content, respectively, when compared to control. In parallel, ROS content was decreased in a concentration-dependent manner. Data also revealed a promising redox potential of Sch-642305, which was isolated from F3_DCM_ and accounts for ~30, 50, and 90% of SOD, CAT and ROS scavenger activities, respectively, exhibited by such fraction (Figure 11C). However, no statistical difference was observed on GPx activity. By other hand, F3_EtAc_ promoted a modest effect on redox parameters when compared to eEtAc, increasing in 60, 270, and 86% SOD and CAT activities and SH content, respectively, when compared to control (Figure 11E). Finally, a comparative analysis of redox activity of eDCM/F3_DCM_/Sch-642305 (10 μg/mL) and eEtAc/F3_EtAc_ (100 μg/mL) was performed (Figures 12A,B). In a general way, F3_DCM_ fraction exhibited higher redox activity when compared to crude extract or to isolated lactone, which could be a result of synergic effect of different molecules enriched in this fraction (Figure 12A). Regarding extracts obtained using EtAc as solvent, eEtAc exhibited higher antioxidant activity when compared to F3_EtAc_, except for CAT activity (Figure 12B). Nonetheless, all treatments decreased in a similar manner ROS levels in glioma cells.

Although already described limitations of DCFH-DA assay applied in the current study (34), this analysis associated to evaluation of the antioxidant non-enzymatic and enzymatic activities could be of some use in providing an indication of a change in the redox state of a cell (35). Therefore, taken together, these data suggest that bioactive metabolites produced by endophytic fungus modulate redox status of glioma cells which in turn, may affect cell cycle and death pathways, resulting in inhibition of glioma progression.

## Discussion

Natural products have played an important role in the development of therapeutic agents. Recent scientific reports have revealed hundreds of secondary metabolites produced from symbiotic microorganisms, especially endophytic fungi, which stand out their pharmacologic properties, especially antitumor activity (36–38). These microorganisms have been recognized as promising source of bioactive compounds that may act to inhibit or regulate the proliferation and cell cycle, being valuable in anticancer drugs discovery (36, 39, 40). Although several medicinal plant species are recognize to harbor many endophytic fungi, there are no reports describing the endophytic composition of *A. satureioides*.

In the present study, we described the isolation of endophytic fungus from *A. satureioides* and explored the cytotoxic activity against glioma cell lines. Endophyte isolated was named as MF31b11. Since an endophyte can produce a complex mixture of compounds, it becomes interesting to extract bioactive compounds using organic solvents in increasing order of polarity (21, 41). Overall, microbial metabolites released into the liquid culture medium by the endophytic fungus are often extracted with DCM and EtAc (21, 42, 43). In this study, DCM and EtAc were employed as solvents of lower and higher polarity, respectively, to obtain the secondary metabolite compounds produced by MF31b11.

Initially, we report antiglioma activity of DCM and EtAc crude extracts of endophyte isolated. The eDCM showed very promising antitumor activity with lowest IC_50_ of 4.0 μg/mL against rat C6 and human U87MG glioblastoma cell lines. This effect suggests that metabolites with antiglioma properties, produced by the endophytic fungus isolated, present low polarity. In contrast, both extracts did not promote cytotoxicity to primary astrocyte cultures. Recently, endophytic fungi have been shown to produce a plethora of new compounds (44, 45). These bioactive metabolites are interesting to development of new medicines. Therefore, to understand the selective antiglioma effect of both crude extracts, we performed a fractionation. F3_EtAc_ and F3_DCM_ fractions showed interesting cytotoxic effects with lower IC_50_ values 2 and 71 μg/mL, respectively. In addition, fractions induced significant inhibition of cell growth by clonogenic test, such as crude extracts. Clonogenic cell survival assay determines cytostatic effects of a cytotoxic agent, by measuring the proliferative ability of a single cell to form a clone and produce a viable colony (27, 46). Therefore, the size and the number of colonies represent indicators of the cell reproductive death (47).

Cell division, differentiation and death are controlled by several mechanisms ensuring tissue homeostasis (48, 49). Cell cycle involves regulation of DNA structure checkpoints, which arrest the cell cycle at the different phases in response to DNA damage or incomplete replication (50). Thus, deregulation of the cell cycle induces aberrant cell proliferation characteristic of cancer and loss of cell cycle checkpoint control promotes genetic instability (51). In this context, the cell cycle machinery represents an alternative target for diagnostic and therapeutic interventions (51). In present study, the cell cycle analysis of cells treated with eDCM (IC_50_ 2.5 μg/mL) and eEtAc (IC_50_ 50 μg/mL) crude extracts exhibited significant increase of apoptotic cell population on sub-G1 phase and decrease of cells on G1 stage. Apoptosis is characterized by several morphological changes, which include cell shrinkage, chromatin condensation, and nuclear fragmentation (52). Moreover, this process of programmed cell death has been recognized as one of the major causes that mediate inhibition of cell proliferation and may be therapeutically exploited for cancer treatment (38, 53). Several studies have been reported that accumulation of cells in sub-G1 phase indicates cell death by apoptosis characterized by DNA fragmentation (54–56). By other hand, F3_EtAc_ fraction induced late apoptosis on glioma cells with increase of cells on G2/M phase and a corresponding decrease of cells in G1 stage. The G2/M checkpoint is a known target for cell cycle inhibition (57). Evidences suggest that numerous anticancer metabolites from endophytic fungi induce apoptotic death and cell cycle arrest at the G2/M phase (38, 58, 59).

Based on promising activity of F3_DCM_ fraction the molecule identified by LC-MS analysis as Sch-642305, a 10-membered macrolide which showed anti-proliferative properties against C6 and U138MG glioma cells, with lower IC_50_ of 1.1 and 7.6 μg/mL, respectively (5 and 30.1 μM). Malignant gliomas are characterized by the diffuse invasion of distant brain tissue due to its migratory capacity, thus compounds with anti-migratory potential becoming interesting (60, 61). In this study, the Sch-642305 significantly decreased the migration of glioma cells. In addition, this molecule induced cell death mainly by apoptosis, accumulation of cells on G2/M stage, promoting alterations on a reproductive ability cells by inhibiting colony formation. Sch-642305 was first isolated from *Penicillium verrucosum* as a bacterial DNA primase inhibitor (62). Moreover, this lactone is described as the major compound produced by the endophytic fungus *Phomopsis* sp. with interesting cytotoxic activity against human colorectal carcinoma (HCT-116), human breast adenocarcinoma (MDA-MB-231) and human myelogenous leukemia (K562) (33, 63). However, there are no reports about the elucidation of its antiglioma activity and its antiproliferative mechanisms.

Several hallmarks of cancer, such as genomic instability, resistance to apoptosis, uncontrolled proliferation and angiogenesis are promoted by the increased ROS levels commonly found in tumor cells (6, 64). Glioblastoma is characterized by high quantities of ROS into the cells, as superoxide anion (${\text{O}}_{2}^{\cdot -}$) and hydrogen peroxide (H_2_O_2_), favoring oxidative environment, cell damage and invasiveness of glioma cells (7, 65). We found that Sch-642305 inhibited oxidative stress, evidenced by increased SOD and CAT enzymes activity and suppression of ROS production. High levels of antioxidant enzymes are required to remove high levels of free radicals to protect against damage to brain tissues (66). SOD is the first line of enzymatic antioxidant defense which metabolizes ${\text{O}}_{2}^{\cdot -}$ to H_2_O_2_ and water (67, 68). However, H_2_O_2_ when accumulated into the cells and tissue is highly toxic. In order to prevent this phenomenon, CAT acts in the detoxification of H_2_O_2_ and consequently reducing the damage induced by free radicals (69). In addition, Sch-642305 promoted increase of sulfhydryl content on glioma cells. This group of thiols consist an important non-enzymatic defense system playing a critical role in oxidative stress, apoptosis, detoxification and cellular signal transmission, and in enzymatic activities (70, 71). These results suggest that antiglioma effect of lactone isolated is due to reduction of ROS levels while increasing antioxidant defense.

Furthermore, eDCM and eEtAc also reduce oxidative damages against glioma cells due to increase of SOD, CAT and GPx antioxidant enzymes consequently, decrease of ROS production. GPx is an important intracellular enzyme that breakdown H_2_O_2_ to water, playing a crucial role of inhibiting lipid peroxidation process, thereby protecting cells from oxidative stress (69). Thus, these enzymes work in conjunction and therefore could be useful to determine the antioxidant status of several compounds (72, 73). Similarly, F3_DCM_ and F3_EtAc_ fractions also showed antioxidant activity against glioma cells. Our study also reveals that the synergism between Sch-642305 and others compounds present in F3_DCM_ fraction induces changes in oxidative parameters like increases enzymatic and non-enzymatic antioxidant defense as well as suppress ROS generation. However, it is important to highlight that the Sch-642305 played an important role to antiproliferative activity and modulation of redox status against glioma cells exhibited by F3_DCM_ fraction.

## Conclusion

The current study is the first reporting about isolation of endophytic fungus from *A. satureioides*. Crude extracts showed cytotoxic activity against glioma cells by inducing apoptosis. Furthermore, F3_DCM_ and F3_EtAc_ fractions exhibited significant selective antiglioma activity and modulated cell redox status. In addition, our data describe structural identification and cytotoxicity of Sch-642305 from F3_DCM_ fraction, which showed promising chemotherapeutic potential. Sch-642305 promoted cell death by apoptosis, decreased glioma cell migration, enhanced antioxidant defense system and suppressed ROS production. These data were summarized in the Supplementary Figure 1. Hence, our study highlights the role of Sch-642305 as a possible therapy for gliomas as well as the importance of endophyte in novel anti-cancer drug discovery, encouraging research in this field.

## Author contributions

NP wrote the paper, endophytic fungus manipulation, cell culture, cytotoxicity and oxidative stress experiments. KG manipulation of endophytic fungus and biomass production. DdS and WC production of organic crude extracts. PTR cell culture and cytotoxicity experiments of crude and fractionated extracts. NB cell culture and cytotoxicity experiments of fractions, cell cycle, cell death and clonogenic analysis. JA cell culture and cell cycle analysis. KC chemical analysis of extracts and molecule elucidation. AS, EdB, and PVR chemical analysis and production of fractionated extracts. MS, RS, and FS oxidative stress experiments. EB advisor, writing, cell culture, and cytotoxicity experiments.

### Conflict of interest statement

The authors declare that the research was conducted in the absence of any commercial or financial relationships that could be construed as a potential conflict of interest.

## Supplementary material

Supplementary Figure 1 Metabolites produced by the endophytic fungus isolated from Achyrocline satureioides inhibit cell proliferation by inducing changes in the cell cycle, apoptosis and by reducing oxidative stress.

## References

[1] Ramirez YP Weatherbee JL Wheelhouse RT Ross AH. Glioblastoma multiforme therapy and mechanisms of resistance. Pharmaceuticals (2013) 6:1475–506. 10.3390/ph6121475 24287492 PMC3873674

[2] Piccirillo SG Sottoriva A Watts C. The role of sub-ventricular zone in gliomagenesis. Aging (Albany NY) (2015) 7:738–9. 10.18632/aging.100823 26527608 PMC4637196

[3] Eder K Kalman B. The dynamics of interactions among immune and Glioblastoma cells. Neuromol Med. (2015) 17:335–52. 10.1007/s12017-015-8362-x 26224516

[4] Gilbert MR Dignam JJ Armstrong TS Wefel JS Blumenthal DT Vogelbaum MA. A randomized trial of Bevacizumab for newly diagnosed Glioblastoma. N Engl J Med. (2014) 370:699–708. 10.1056/NEJMoa1308573 24552317 PMC4201043

[5] Hecht F Cazarin JM Lima CE Faria CC Da Costa Leitão AA Ferreira AC. Redox homeostasis of breast cancer lineages contributes to differential cell death response to exogenous hydrogen peroxide. Life Sci. (2016) 158:7–13. 10.1016/j.lfs.2016.06.016 27328417

[6] Ciccarese F Ciminale V. Escaping death: mitochondrial redox homeostasis in cancer cells. Front Oncol. (2017) 7:117. 10.3389/fonc.2017.00117 28649560 PMC5465272

[7] Salazar-Ramiro A Ramírez-Ortega D Pérez de la Cruz V Hérnandez-Pedro NY González-Esquivel DF Sotelo J. Role of redox status in development of glioblastoma. Front Immunol. (2016) 7:156. 10.3389/fimmu.2016.00156 27199982 PMC4844613

[8] Retta D Dellacassa E Villamil J Suárez SA Bandoni AL Marcela, a promising medicinal and aromatic plant from Latin America: a review. Ind Crops Prod. (2012) 38:27–38. 10.1016/j.indcrop.2012.01.006

[9] Silva LM Farias JAM Boeing T Somensi LB Beber AP Cury BJ. Hydroalcoholic extract from inflorescences of *Achyrocline satureioides* (Compositae) Ameliorates Dextran Sulphate sodium-induced colitis in mice by attenuation in the production of inflammatory cytokines and oxidative mediators. J Evid Complement Altern Med. (2016) 2016:3475356. 10.1155/2016/3475356 27847525 PMC5099481

[10] Yamane LT De Paula E Jorge MP De Freitas-Blanco VS Junior ÍM Figueira GM. *Acmella oleracea* and *Achyrocline satureioides* as sources of natural products in topical wound care. J Evid Based Complement Altern Med. (2016) 2016:1–9. 10.1155/2016/3606820 27777596 PMC5061968

[11] Kusari S Singh S Jaybaskaran C. Biotechnological potential of plant-associated endophytic fungi: hope versus hype. Trends Biotechnol. (2014) 32:297–303. 10.1016/j.tibtech.2014.03.009 24703621

[12] Strobel GA Long DM Endophytic microbes embody pharmaceutical potential. ASM News (1998) 64:263–8.

[13] Kusari S Spiteller M Metabolomics of endophytic fungi producing associated plant secondary metabolites: progress, challenges and opportunities. In: Roessner U editor. Metabolomics. Rijeka, Croatia: InTech (2012). p. 241–66.

[14] Kusari S Hertweck C Spiteller M. Chemical ecology of endophytic fungi: origins of secondary metabolites. Chem Biol. (2012) 19:792–8. 10.1016/j.chembiol.2012.06.004 22840767

[15] Cheng L Zhang QY Jia M Ming QL Yue W Rahman K Endophytic fungi with antitumor activities: their occurrence and anticancer compounds. Crit Rev Microbiol. (2014) 42:454–73. 10.3109/1040841X.2014.95989 25343583

[16] Carini JP Klamt F Bassani VL Flavonoids from *Achyrocline satureioides*: promising biomolecules for anticancer therapy. RSC Adv. (2014) 4:3131–44. 10.1039/C3RA43627F

[17] Salgueiro AC Folmer V Da Rosa HS Costa MT Boligon AA Paula FR. *In vitro* and *in silico* antioxidant and toxicological activities of *Achyrocline satureioides*. J Ethnopharmacol. (2016) 194:6–14. 10.1016/j.jep.2016.08.048 27575777

[18] Moresco KS Silveira AK Schnorr CE Zeidán-Chuliá F Bortolin RC Bittencourt LDS. Supplementation with *Achyrocline satureioides* inflorescence extracts to pregnant and breastfeeding rats induces tissue-specific changes in enzymatic activity and lower neonatal survival. Biomedicines (2017) 5:53. 10.3390/biomedicines5030053 29093434 PMC5618311

[19] Bertozzo F Machado IS Meios de cultura no desenvolvimento de ápices caulinares de mamoneira (*Ricinus communis* L.) *in vitro*. Ciênc Agrotec. (2010) 34:1477–82. 10.1590/S1413-70542010000600018

[20] Rocha R Luz DED Engels C Pileggi SAV Jaccoud Filho DDS Matiello RR. Selection of endophytic fungi from comfrey (*Symphytum officinale* L.) for *in vitro* biological control of the phytopathogen *Sclerotinia sclerotiorum* (Lib.). Braz J Microbiol. (2009) 40:73–8. 10.1590/S1517-83822009000100011 24031320 PMC3768515

[21] Seidel V. Initial and bulk extraction of natural products isolation. Methods Mol Biol. (2012) 864:27–41. 10.1007/978-1-61779-624-1_2 22367892

[22] Aguiar Galvão WR Braz Filho R Canuto KM Ribeiro PRV Campos AR Moreira ACOM Gastroprotective and anti-inflammatory activities integrated to chemical composition of *Myracrodruon urundeuva* Allemão—A conservationist proposal for the species. J Ethnopharmacol. (2018) 222:177–89. 10.1016/j.jep.2018.04.024 29689352

[23] Da Frota MLJ Braganhol E Canedo AD Klamt F Apel MA Mothes B Brazilian marine sponge *Polymastia janeirensis* induces apoptotic cell death in human U138MG glioma cell line, but not in a normal cell culture. Invest New Drugs (2009) 27:13–20. 10.1007/s10637-008-9134-3 18454276

[24] Mosmann T. Rapid colorimetric assay for cellular growth and survival: application to proliferation and cytotoxicity assays. J Immunol Methods (1983) 65:55–63. 10.1016/0022-1759(83)90303-4 6606682

[25] Pauwels B Korst AE De Pooter CM Pattyn GG Lambrechts HA Baay MF. Comparison of the sulforhodamine B assay and the clonogenic assay for *in vitro* chemoradiation studies. Cancer Chemother Pharmacol. (2003) 51:221–6. 10.1007/s00280-002-0557-9 12655440

[26] Kaczmarek E Erb L Koziak K Jarzyna R Wink MR Guckelberger O. Modulation of endothelial cell migration by extracellular nucleotides. Involvement of focal adhesion kinase and phosphatidylinositol 3-kinase-mediated pathways. Thromb Haemost. (2005) 93:735–42. 10.1267/THRO05040735 15841322 PMC2830093

[27] Franken NAP Rodermond HM Stap J Haveman J Bree CV. Clonogenic assay of cells *in vitro*. Nat Protoc. (2006) 1:2315–9. 10.1038/nprot.2006.339 17406473

[28] Viau CM Moura DJ Facundo VA Saffi J. The natural triterpene 3β,6β,16β-trihydroxy-lup-20(29)-ene obtained from the flowers of Combretum leprosum induces apoptosis in MCF-7 breast cancer cells. BMC Complement Altern Med. (2014) 14:280. 10.1186/1472-6882-14-280 25086656 PMC4129108

[29] Dos Santos LM Da Silva TM Azambuja JH Ramos PT Oliveira PS Silveira EF. Methionine and methionine sulfoxide treatment induces M1/classical macrophage polarization and modulates oxidative stress and purinergic signaling parameters. Mol Cell Biochem. (2017) 424:69–78. 10.1007/s11010-016-2843-6 27752805

[30] Misra HP Fridovich I. The role of superoxide anion in the autoxidation of epinephrine and a simple assay for superoxide dismutase. J Biol Chem. (1972) 247:3170–5. 4623845

[31] Aebi H. Catalase *in vitro*. Methods Enzymol. (1984) 105:121–6. 10.1016/S0076-6879(84)05016-3 6727660

[32] Aksenov MY Markesbery WR. Changes in thiol content and expression of glutathione redox system genes in the hippocampus and cerebellum in Alzheimer's disease. Neurosci Lett. (2001) 302:141–5. 10.1016/S0304-3940(01)01636-6 11290407

[33] Adelin E Servy C Cortial S Lévaique H Martin TM Retailleau P. Isolation, structure elucidation and biological activity of metabolites from Sch-642305-producing endophytic fungus *Phomopsis* sp. CMU-LMA. Phytochemistry (2011a) 72:2406–12. 10.1016/j.phytochem.2011.08.010 21924749

[34] Kalyanaraman B Darley-Usmar V Davies KJ Dennery PA Forman HJ Grisham MB. Measuring reactive oxygen and nitrogen species with fluorescent probes: challenges and limitations. Free Radical Bio Med. (2012) 52:1–6. 10.1016/j.freeradbiomed.2011.09.030 22027063 PMC3911769

[35] Winterbourn CC. The challenges of using fluorescent probes to detect and quantify specific reactive oxygen species in living cells. Biochim Biophys Acta (2014) 1840:730–8. 10.1016/j.bbagen.2013.05.004 23665586

[36] Koul M Meena S Kumar A Sharma PR Singamaneni V Hassan S. Secondary metabolites from endophytic fungus *Penicillium pinophilum* induce ROS-mediated apoptosis through mitochondrial pathway in pancreatic cancer cells. Planta Med. (2016) 82:344–55. 10.1055/s-0035-1558308 26848704

[37] Uesugi S Fujisawa N Yoshida J Watanabe M Dan S Yamori T. Pyrrocidine A, a metabolite of endophytic fungi, has a potent apoptosis-inducing activity against HL60 cells through caspase activation via the Michael addition. J Antibiot. (2016) 69:133–40. 10.1038/ja.2015.103 26506860

[38] Wang F Jiang J Hu S Ma H Zhu H Tong Q. Secondary metabolites from endophytic fungus *Chaetomium* sp. induce colon cancer cell apoptotic death. Fitoterapia (2017) 121:86–93. 10.1016/j.fitote.2017.06.021 28652012

[39] Chandra S. Endophytic fungi: novel sources of anticancer lead molecules. Appl Microbiol Biotechnol. (2012) 95:47–59. 10.1007/s00253-012-4128-7 22622838

[40] Fatima N Kondratyuk TP Park EJ Marler LE Jadoon M Qazi MA. Endophytic fungi associated with *Taxusfuana* (West Himalayan Yew) of Pakistan: potential bio-resources for cancer chemopreventive agents. Pharm Biol. (2016) 54:2547–54. 10.3109/13880209.2016.1170154 27159021

[41] Zin NM Remali J Nasrom MN Ishak SA Baba MS Jalil J Bioactive compounds fractionated from endophyte Streptomyces SUK 08 with promising *ex-vivo* antimalarial activity. Asian Pac J Trop Biomed. (2017) 7:1062–6. 10.1016/j.apjtb.2017.10.006

[42] Yadav M Yadav A Yadav JP *In vitro* antioxidant activity and total phenolic content of endophytic fungi isolated from *Eugenia jambolana* Lam. Asian Pac J Trop Med. (2014) 7:S256–61. 10.1016/S1995-7645(14)60242-X 25312132

[43] Jinfeng EC Rafi MIM Hoon KC Lian HK Kqueen CY. Analysis of chemical constituents, antimicrobial and anticancer activities of dichloromethane extracts of *Sordariomycetes* sp. endophytic fungi isolated from *Strobilanthes crispus*. World J Microb Biotechnol. (2017) 33:5. 10.1007/s11274-016-2175-4 27844243

[44] Li G Kusari S Lamshoft M Schuffler A Laatsch H Spiteller M Antibacterial Secondary metabolites from an endophytic fungus, *Eupenicillium* sp. LG41. J Nat Prod. (2014) 11:2335–41. 10.1021/np500111w 25356913

[45] Jia M Chen L Xin HL Zheng CJ Rahman K Han T. A friendly relationship between endophytic fungi and medicinal plants: a systematic review. Front Microbiol. (2016) 7:1–14. 10.3389/fmicb.2016.00906 27375610 PMC4899461

[46] Sumantran VN. Cellular chemosensitivity assays: an overview. In: Cree EA, editor. Cancer Cell Culture. Portsmouth: Humana Press (2011). p. 219–36. 10.1007/978-1-61779-080-5_19 21516411

[47] Miyashita T Higuchi Y Kojima M Ochiai A Ishii G. Single cell time-lapse analysis reveals that podoplanin enhances cell survival and colony formation capacity of squamous cell carcinoma cells. Sci Rep. (2017) 7:39971. 10.1038/srep39971 28059107 PMC5216406

[48] Hanahan D Weinberg RA. Hallmarks of cancer: the next generation. Cell (2011) 144:646–74. 10.1016/j.cell.2011.02.013 21376230

[49] Wiman KG Zhivotovsky B. Understanding cell cycle and cell death regulation provides novel weapons against human diseases. J Intern Med. (2017) 281:483–95. 10.1111/joim.12609 28374555

[50] Bertoli C Skotheim JM De Bruin RA. Control of cell cycle transcription during G1 and S phases. Nat Rev Mol Cell Biol. (2013) 14:518–28. 10.1038/nrm3629 23877564 PMC4569015

[51] Williams GH Stoeber K. The cell cycle and cancer. J Pathol. (2012) 226:352–64. 10.1002/path.3022 21990031

[52] Walker AM Stevens JJ Ndebele K Tchounwou PB Arsenic trioxide modulates DNA synthesis and apoptosis in lung carcinoma cells. Int J Environ Res Public Health (2010) 7:1996–2007. 10.3390/ijerph7051996 20632473 PMC2864039

[53] Bai L Wang S. Targeting apoptosis pathways for new cancer therapeutics. Annu Rev Med. (2014) 65:139–55. 10.1146/annurev-med-010713-141310 24188661

[54] Ahmad J Alhadlaq HA Siddiqui MA Saquib Q Al-Khedhairy AA Musarrat J. Concentration dependent induction of reactive oxygen species, cell cycle arrest and apoptosis in human liver cells after nickel nanoparticles exposure. Environ Toxicol. (2015) 30:137–48. 10.1002/tox.21879 23776134

[55] Vessoni AT Quinet A Andrade-Lima LC Martins DJ Garcia CCM Rocha CRR Chloroquine-induced glioma cells death is associated with mitochondrial membrane potential loss, but not oxidative stress. Free Rad Biol Med. (2016) 90:91–100. 10.1016/j.freeradbiomed.2015.11.008 26577174

[56] Agrawal S Chaugule S More S Rane G Indap M. Methanolic extract of *Euchelus asper* exhibits in-ovo anti-angiogenic and *in vitro* anti-proliferative activities. Biol Res. (2017) 50:41. 10.1186/s40659-017-0147-2 29233192 PMC5726033

[57] Newell M Baker K Postovit LM Field CJ. A critical review on the effect of docosahexaenoic acid (DHA) on cancer cell cycle progression. Int J Mol Sci. (2017) 18:1784. 10.3390/ijms18081784 28817068 PMC5578173

[58] Wang FQ Tong QY Ma HR Xu HF Hu S Ma W. Indole diketopiperazines from endophytic *Chaetomium* sp. 88194 induce breast cancer cell apoptotic death. Sci Rep. (2015) 5:9294. 10.1038/srep09294 25787158 PMC4365412

[59] Pathania AS Guru SK Ashraf NU Riyaz-Ul-Hassan S Ali A Tasduq SA. A novel stereo bioactive metabolite isolated from an endophytic fungus induces caspase dependent apoptosis and STAT-3 inhibition in human leukemia cells. Eur J Pharmacol. (2015) 765:75–85. 10.1016/j.ejphar.2015.08.018 26291658

[60] Giese A Bjerkvig R Berens ME Westphal M. Cost of migration: invasion of malignant gliomas and implications for treatment. J Clin Oncol. (2003) 21:1624–36. 10.1200/JCO.2003.05.063 12697889

[61] Lefranc F Brotchi J Kiss R. Possible future issues in the treatment of glioblastomas: special emphasis on cell migration and the resistance of migrating glioblastoma cells to apoptosis. J Clin Oncol. (2005) 23:2411–22. 10.1200/JCO.2005.03.089 15800333

[62] Chu M Mierzwa R Xu L He L Terracciano J Patel M. Isolation and structure elucidation of Sch 642305, a novel bacterial DNA primase inhibitor produced by *Penicillium verrucosum*. J Nat Prod. (2003) 66:1527–30. 10.1021/np0302302 14695789

[63] Adelin E Servy C Cortial S Lévaique H Gallard F Martin MT. Biotransformation of natural compounds. Oxido-reduction of Sch-642305 by *Aspergillus ochraceus* ATCC 1009. Bioorganic Med Chem Lett. (2011b) 21:2456–9. 10.1016/j.bmcl.2011.02.063 21396813

[64] Morry J Ngamcherdtrakul W Yantasee W. Oxidative stress in cancer and fibrosis: opportunity for therapeutic intervention with antioxidant compounds, enzymes, and nanoparticles. Redox Biol. (2017) 11:240–53. 10.1016/j.redox.2016.12.011 28012439 PMC5198743

[65] Feng J Yan PF Zhao HY Zhang FC Zhao WH Feng M. SIRT6 suppresses glioma cell growth via induction of apoptosis, inhibition of oxidative stress and suppression of JAK2/STAT3 signaling pathway activation. Oncol Rep. (2016) 35:1395–402. 10.3892/or.2015.4477 26648570

[66] Martínez-Martos JM Mayas MD Carrera P De Saavedra JMA Sánchez-Agesta R Arrazola M Phenolic compounds oleuropein and hydroxytyrosol exert differential effects on glioma development via antioxidant defense systems. J Funct Foods (2014) 11:221–34. 10.1016/j.jff.2014.09.006

[67] Zhou J Li Y Yan G Bu Q Lv L Yang Y. Protective role of taurine against morphine-induced neurotoxicity in C6 cells via inhibition of oxidative stress. Neurotox Res. (2011) 20:334. 10.1007/s12640-011-9247-x 21611853

[68] Schieber M Chandel NS. ROS function in redox signaling and oxidative stress. Curr Biol. (2014) 24:453–62. 10.1016/j.cub.2014.03.034 24845678 PMC4055301

[69] Ighodaro OM Akinloye OA First line defence antioxidants-superoxide dismutase (SOD), catalase (CAT) and glutathione peroxidase (GPX): their fundamental role in the entire antioxidant defence grid. Alexandria J Med. (2017):1–7. 10.1016/j.ajme.2017.09.001

[70] Ergin M Caliskanturk M Senat A Akturk O Erel O. Disulfide stress in carbon monoxide poisoning. Clin Biochem. (2016) 49:1243–7. 10.1016/j.clinbiochem.2016.07.019 27497239

[71] Simşek E Erel O Bicer CK Çarlioglu A. A novel method for determining the relation between nasal polyposis and oxidative stress: the thiol/disulphide homeostasis. Acta Oto-laryngol. (2016) 136:1180–3. 10.1080/00016489.2016.1186833 27222940

[72] Lowe F Biomarkers of oxidative stress. In: Heidelberg GER, editor. Systems Biology of Free Radicals and Antioxidants. Vol. 2 Berlin; Heidelberg: Springer (2014). p. 65–87.

[73] Del Bó C Martini D Porrini M Klimis-Zacas D Riso P Berries and oxidative stress markers: an overview of human intervention studies. Food Funct. (2015) 6:2890–917. 10.1039/c5fo00657k 26226324

