# Supplementary figures and images for: Endophytic Fungus Isolated From Achyrocline satureioides Exhibits Selective Antiglioma Activity—The Role of Sch-642305
Source: Front Oncol. 2018 Oct 29;8:476. doi: 10.3389/fonc.2018.00476 (PMC6215846; doi:10.3389/fonc.2018.00476)

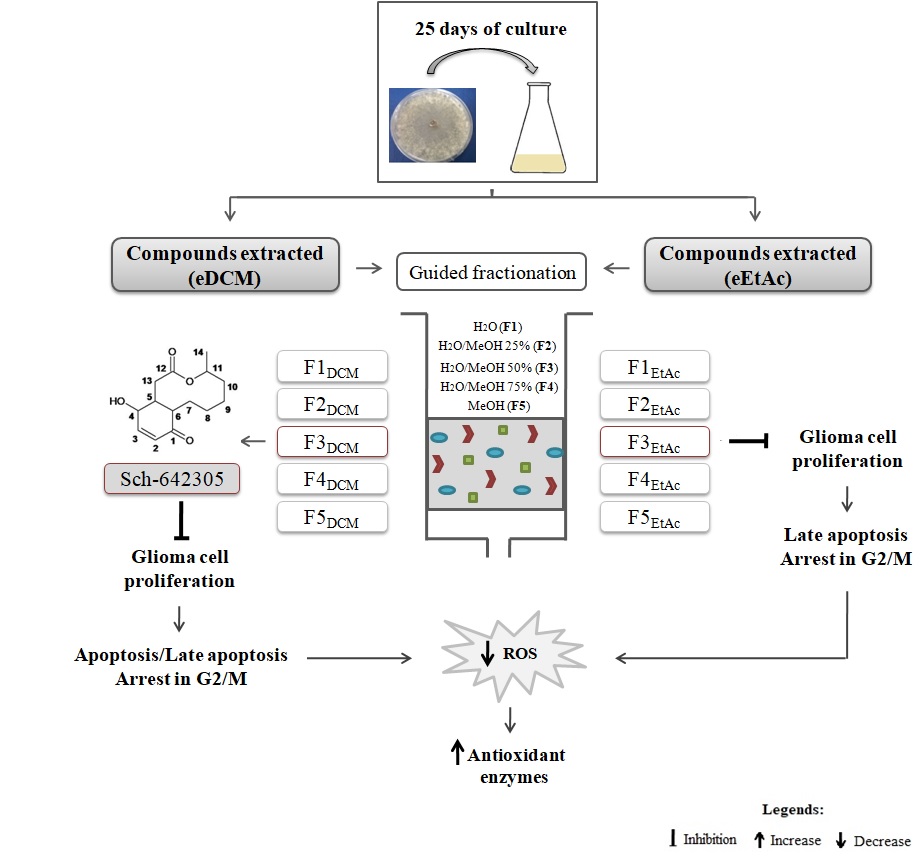

Supplement: Supplementary Figure 1 — Metabolites produced by the endophytic fungus isolated from Achyrocline satureioides inhibit cell proliferation by inducing changes in the cell cycle, apoptosis and by reducing oxidative stress. [file Image_1.JPEG]
